# Network Analyses Reveal Novel Aspects of ALS Pathogenesis

**DOI:** 10.1371/journal.pgen.1005107

**Published:** 2015-03-31

**Authors:** Mario Sanhueza, Andrea Chai, Colin Smith, Brett A. McCray, T. Ian Simpson, J. Paul Taylor, Giuseppa Pennetta

**Affiliations:** 1 Centre for Integrative Physiology, University of Edinburgh, Edinburgh, United Kingdom; 2 Euan MacDonald Centre for Motor Neuron Disease Research, University of Edinburgh, Edinburgh, United Kingdom; 3 Department of Molecular and Human Genetics, Baylor College of Medicine, Jan and Dan Duncan Neurological Research Institute, Houston, Texas, United States of America; 4 Academic Department of Neuropathology, Centre for Clinical Brain Sciences, University of Edinburgh, Edinburgh, United Kingdom; 5 Department of Neurology, Massachusetts General Hospital, Harvard Medical School, Cambridge, Massachusetts, United States of America; 6 Biomathematics and Statistics Scotland, University of Edinburgh, United Kingdom; 7 Institute for Adaptive and Neural Computation, School of Informatics, University of Edinburgh, United Kingdom; 8 Department of Cell and Molecular Biology, St. Jude Children’s Research Hospital, Memphis, Tennessee, United States of America; Stanford University School of Medicine, UNITED STATES

## Abstract

Amyotrophic Lateral Sclerosis (ALS) is a fatal neurodegenerative disease characterized by selective loss of motor neurons, muscle atrophy and paralysis. Mutations in the human VAMP-associated protein B (hVAPB) cause a heterogeneous group of motor neuron diseases including ALS8. Despite extensive research, the molecular mechanisms underlying ALS pathogenesis remain largely unknown. Genetic screens for key interactors of hVAPB activity in the intact nervous system, however, represent a fundamental approach towards understanding the in vivo function of hVAPB and its role in ALS pathogenesis. Targeted expression of the disease-causing allele leads to neurodegeneration and progressive decline in motor performance when expressed in the adult *Drosophila*, eye or in its entire nervous system, respectively. By using these two phenotypic readouts, we carried out a systematic survey of the *Drosophila* genome to identify modifiers of hVAPB-induced neurotoxicity. Modifiers cluster in a diverse array of biological functions including processes and genes that have been previously linked to hVAPB function, such as proteolysis and vesicular trafficking. In addition to established mechanisms, the screen identified endocytic trafficking and genes controlling proliferation and apoptosis as potent modifiers of ALS8-mediated defects. Surprisingly, the list of modifiers was mostly enriched for proteins linked to lipid droplet biogenesis and dynamics. Computational analysis reveals that most modifiers can be linked into a complex network of interacting genes, and that the human genes homologous to the *Drosophila* modifiers can be assembled into an interacting network largely overlapping with that in flies. Identity markers of the endocytic process were also found to abnormally accumulate in ALS patients, further supporting the relevance of the fly data for human biology. Collectively, these results not only lead to a better understanding of hVAPB function but also point to potentially relevant targets for therapeutic intervention.

## Author Summary

Amyotrophic Lateral Sclerosis (ALS) is a neurodegenerative disease causing loss of motor neurons and consequently a progressive deterioration of motor functions. ALS is uniformly fatal with death occurring 5 years after onset of symptoms. There is currently no effective treatment for ALS. Several mutations in a gene called hVAPB have shown that this gene is causative of a type of ALS known as ALS8. In this study we sought to identify genes and cellular processes that are involved in the toxicity conferred by the defective ALS8 allele. By using the power of *Drosophila* genetics, we performed a large scale genomic screen and identified a number of genes that can affect hVAPB-mediated toxicity. These modifiers cluster into functional pathways known to be involved in ALS as well as novel ones. The relevance of these modifiers and mechanisms for the human disease was confirmed by showing that the human homologues of the fly modifiers can be organized into a network that closely resembles that of the *Drosophila* genes. Identifying cellular processes and proteins that modulate hVAPB pathological activity can facilitate the discovery of an effective treatment for ALS.

## Introduction

Amyotrophic lateral sclerosis (ALS) is characterized by the degeneration of upper and lower motor neurons leading to a progressive deterioration of motor function and ultimately death, due to respiratory failure. Both environmental and genetic factors have been shown to contribute to ALS susceptibility. Approximately 10% of patients with ALS have a family history while the majority of ALS cases are classified as sporadic [1]. In rare inherited familial forms of ALS, specific mutations in causative genes have been identified and this has significantly advanced the field [2]. However, the mechanisms by which these mutations cause motor neuron dysfunction and death remain unknown.

Several mutations in the human VAMP-associated protein B (hVAPB) have been identified as being causative of a type of ALS known as ALS8 [3–5]. hVAPB is a member of a highly conserved protein family involved in a variety of functions including maintenance of endoplasmic reticulum (ER) morphology, vesicular trafficking and intracellular lipid transport [6]. In *C*.*elegans*, the N-terminal domain of the protein is cleaved, secreted and functions as an extracellular ligand for Ephrin receptors [7]. Secreted fragments also bind to the leukocyte antigen-related (LAR) and roundabout (Robo) family receptors situated on the muscle cell surface to control actin organization and mitochondrial morphology and function [8]. *Drosophila* Vap-33-1, (hereafter referred as DVAP) controls synaptic remodeling and composition of post-synaptic glutamate receptors [9,10].

The DVAP protein shows only 22% identity with its human orthologue hVAPB but the two proteins share a common structural organization including a transmembrane domain, a coiled-coil domain and a N-terminal region, which has a high degree of similarity to the *C*.*elegans* major sperm proteins (MSP domain). More importantly, expression of hVAPB in flies can rescue the mutant phenotype associated with *DVAP* loss-of-function mutations, indicating that DVAP and hVAPB are functionally interchangeable [10]. DVAP also shows conservation of residues linked to ALS8 in humans (human P56 is equivalent to fly P58, human T46 is equivalent to fly T48 and human V260 is equivalent to fly V264) and in *Drosophila*, expression of transgenic alleles carrying pathogenic mutations reproduces major hallmarks of the human disease, including aggregate formation, chaperon up-regulation and locomotion defects [4,10,11]. A number of studies have implicated the disease-linked alleles in abnormal unfolded protein response (UPR) and in the disruption of both the anterograde axonal transport of mitochondria [12] and calcium homeostasis [13]. Moreover, the P56S disease-linked mutation is a loss-of-function allele through a dominant negative mechanism as it antagonizes the endogenous protein function by recruiting hVAPB into cytoplasmic aggregates [14–16]. Consistent with this disease mechanism, hVAPB levels are decreased in a number of sporadic cases as well as in TDP-43 (transactive response DNA-binding protein 43) and in SOD1 (superoxide dismutase 1) models for ALS [17–19]. The ALS causative gene fused in sarcoma (FUS) has been shown to bind VAPB mRNA and TDP-43 is associated with VAPB-positive aggregates in a transgenic mouse model expressing the P56S pathogenic allele [20–22]. Taken together, these data underline the importance of VAPB in ALS pathogenesis and explain why, in recent years, much effort has been devoted to generate models of ALS8 in a number of experimental organisms including *Drosophila* [2]. Despite this, the molecular mechanisms and the cellular processes underlying ALS8 pathogenesis remain elusive.

Over the last few years, genome-wide genetic screens in yeast have proven to be extremely powerful in identifying novel genetic risk factors for ALS [23–25] and investigating fundamental pathogenic mechanisms associated with the ALS disease proteins TDP-43 and FUS [26–29]. These studies have led to the identification of important genetic modifiers of FUS and TDP-43 toxicity, including RNA binding proteins and proteins involved in RNA metabolism, ribosome biogenesis and cellular stress responses.

To gain insight into fundamental pathogenic aspects of hVAPB-mediated ALS, we adopted a similar approach and by exploiting the power of *Drosophila* genetics, we conducted a genetic screen for modifiers of DVAP-P58S-dependent phenotypes.

From the identified modifiers, a computational network of highly interconnected genes was assembled that encompasses a wide range of functional categories associated with DVAP-P58S toxicity. The *Drosophila* data were evaluated for their relevance to human biology by mapping the *Drosophila* genes to their human homologs and showing that the human genes could be integrated into a network of interacting genes largely overlapping with the *Drosophila* one. Some modifiers clustered within cellular processes that were already known to affect hVAPB protein functions such as proteolysis, intercellular signaling pathways mediated by Robo receptors and phosphoinositide metabolism [4,8,15]. However, we also identified a number of genes and processes that have not been previously associated with hVAPB function. Modifiers included genes involved in lipid droplet (LD) biogenesis and dynamics as well as endocytosis. We further confirmed the role of endocytosis in ALS pathogenesis by showing that the RAB5 GTPase, an early endosomal marker, is altered in *post-mortem* tissues of ALS patients. Importantly, a considerable number of DVAP-P58S modifiers are members of the Ras and Hippo (Hpo) signal transduction pathways, which control cell proliferation and apoptosis. Our data point to a mechanistic link between Ras and Hpo signaling on one hand and DVAP-P58S-mediated ALS8 phenotypes on the other. These results identify genes and cellular processes that widen our understanding of VAP protein function and provide novel therapeutic targets for ALS and perhaps other closely related neurodegenerative diseases such as fronto-temporal dementia.

## Results

### A large-scale screen in *Drosophila* identifies modifiers of the *DVAP-P58S*-induced eye phenotype

To identify genes that influence DVAP-P58S-mediated pathogenesis, we undertook a genetic modifier screen and looked for genes that upon overexpression suppress or enhance the DVAP-P58S neurodegenerative phenotype in the eye.

Flies, in which the expression of the *DVAP-P58S* transgene under the control of the yeast upstream activating sequence (UAS) was induced by the *eyeless-GAL4* (*ey-GAL4*) driver [30], exhibit a robust eye phenotype characterized by decreased size, fused ommatidia, missing and occasionally supernumerary, bristles [15]. A transgenic strain (*DVAP-P58S* ^*11*^) was identified, whose targeted expression in the eye displays a reduction in eye size to about 30% of the wild-type value making this intermediate phenotype particularly suitable for an enhancer/suppressor screen. This eye phenotype was observed when flies expressing one copy of the *DVAP-P58S* transgene (*ey*,*DVAP-P58S)*, were raised at 30°C to maximize the expression of the transgene (S1F, G, H Fig). A similar phenotype was also associated with the expression of a double copy of the same transgene in the eyes of flies (*ey*,*DVAP-P58S/DVAP-P58S)* raised at 25°C (S1A, C, H Fig). At 28°C flies expressing one DVAP-P58S copy exhibited a phenotype that was less severe than that associated with flies of the same genotype raised at 30°C (S1A, C, H Fig). However, at 28°C and at 30°C flies carrying a double copy of DVAP-P58S failed to hatch possibly due to a strong ectopic expression of the transgene outside the eye. Control flies raised at any of these temperatures did not show any visible eye phenotype (S1A, D, F, H Fig). Taken together, these data clearly indicate that the reduction in eye size results from *DVAP-P58S* expression, it is sensitive to its dosage and it is not affected by the increase in the temperature (S1 Fig). Additionally, we tested whether other disease features, such as the accumulation of aggregates, were dependent on the dosage of the pathogenic transgene (S1I, J, K, L, M Fig). Stainings of larval eye imaginal discs with an anti-DVAP antibody showed a similar accumulation of aggregates positive to DVAP-immuno-reactivity in both *ey*,*DVAP-P58S* flies raised at 30°C and in *ey*,*DVAP-P58S/DVAP-P58S* flies raised at 25°C (S1M, K Fig). A similar accumulation of aggregates was observed in eye imaginal discs of *ey*,*DVAP-P58S* flies raised at 28°C (S1L Fig) and only a few, sporadic aggregates were present in flies of the same genotype raised at 25°C, which exhibit only a small reduction in eye size (S1J, B Fig). Thus, these data show that the extent of inclusion accumulation strictly correlates with the expression levels of the pathogenic transgene and with the severity of the eye phenotype.

For simplicity, we decided to use as tester line for the screen, flies carrying a single copy of the *DVAP-P58S* transgene raised at 30°C.

A line is considered to be a suppressor if it ameliorates the DVAP-P58S phenotype making the DVAP-P58S eye size becoming closer to that of the wild-type. By contrast, a line is an enhancer if it induces a decrease in the eye size of the *ey-Gal4*,*DVAP-P58S* tester line. We previously showed that the reduction in size is due to DVAP-P58S-mediated perturbations in cell survival as coexpression of the *Drosophila* inhibitor of apoptosis 1 (*DIAP1*) transgene substantially rescues the small eye phenotype, with adult flies displaying nearly normal eye size [15].

For the actual screen, *ey-Gal4*,*DVAP-P58S* flies were crossed to mutants selected from publically available collections. As part of a large-scale gene disruption project, the Berkeley *Drosophila* genome project (BDGP) generated thousands of mutations associated with stable insertions of modified P-element transposons [31]. We focused on the screening of the EP and the EPgy2 collections of genome-wide insertional mutations for dominant modifications of the eye neurodegenerative phenotype associated with the *ey-GAL4*,*DVAP-P58S* strain. These collections consist of UAS elements inserted in the promoters of endogenous genes and therefore they can be used to overexpress the downstream gene if the UAS-binding transcription factor GAL4 is co-expressed [32]. These collections were chosen for several reasons. Both collections were generated by random insertions of P-elements throughout the fly genome thus representing a large, unbiased, genome-spanning set of mutant lines. Additionally, both the EP and the EPgy2 collections comprise mutant lines in largely non-overlapping genes in which location and orientation of P-element insertions were previously mapped making the identification of the affected gene relatively straightforward.

We initially focused on the second and the third chromosome and selected 1,120 P-element insertions that, based on the position and orientation of P-elements, are predicted to generate a potential overexpression of the affected gene. For 16 genes for which no EP or EPgy2 insertion lines with the potential of inducing overexpression were available, lines from the P{Mae-UAS.6.11} collection were selected [33,34]. In addition, 47 EP and EPgy2 lines with P-elements inserted in the opposite direction, and therefore with the potential of disrupting the affected genes, were analyzed. As a result, a total of 1,183 lines were tested (S2 Fig).

While performing our screen, we realized that at 30°C, the temperature used to raise flies for the detection of suppressors, the identification of enhancers was difficult as the enhancing effect on the eye phenotype was too subtle. We noticed, however, that flies expressing *DVAP-P58S* under the control of the *ey-GAL4* line exhibit decreased viability possibly due to the ectopic expression of the transgene in brain areas other than the eye. Although not carefully quantified, at 30°C expression of *DVAP-P58S* in the eye results in organism lethality and this effect is sensitive towards genetic modification as enhancers consistently lead to a decrease in the number of eclosed flies. Subsequently, the enhancing effect on the *DVAP-P58S* eye neurodegenerative phenotype was confirmed by rearing flies at 28°C to make the *DVAP-P58S* eye phenotype less severe. In this condition, scoring a putative enhancement of the eye phenotype becomes much easier. At 28°C, DVAP-P58S flies also exhibit partial organism lethality with 80% of the expected number of flies, eclosing. The enhancement effect of modifiers was confirmed by assessing the increase in organism lethality at 28°C.

To eliminate false positives, all interacting insertions were retested two more times by an experimenter who was blind to the identity of the gene. Since we are interested in genes that primarily modify the *DVAP-P58S*-dependent phenotype, lines exhibiting an eye phenotype when driven by the *ey-GAL4* line in the absence of the *DVAP-P58S* transgene were discarded. In summary, only those lines that did not show a neurodegenerative phenotype in *trans* with *ey-GAL4* and for which results could be consistently repeated were designated as modifiers (S2 Fig).

**Fig 1:**
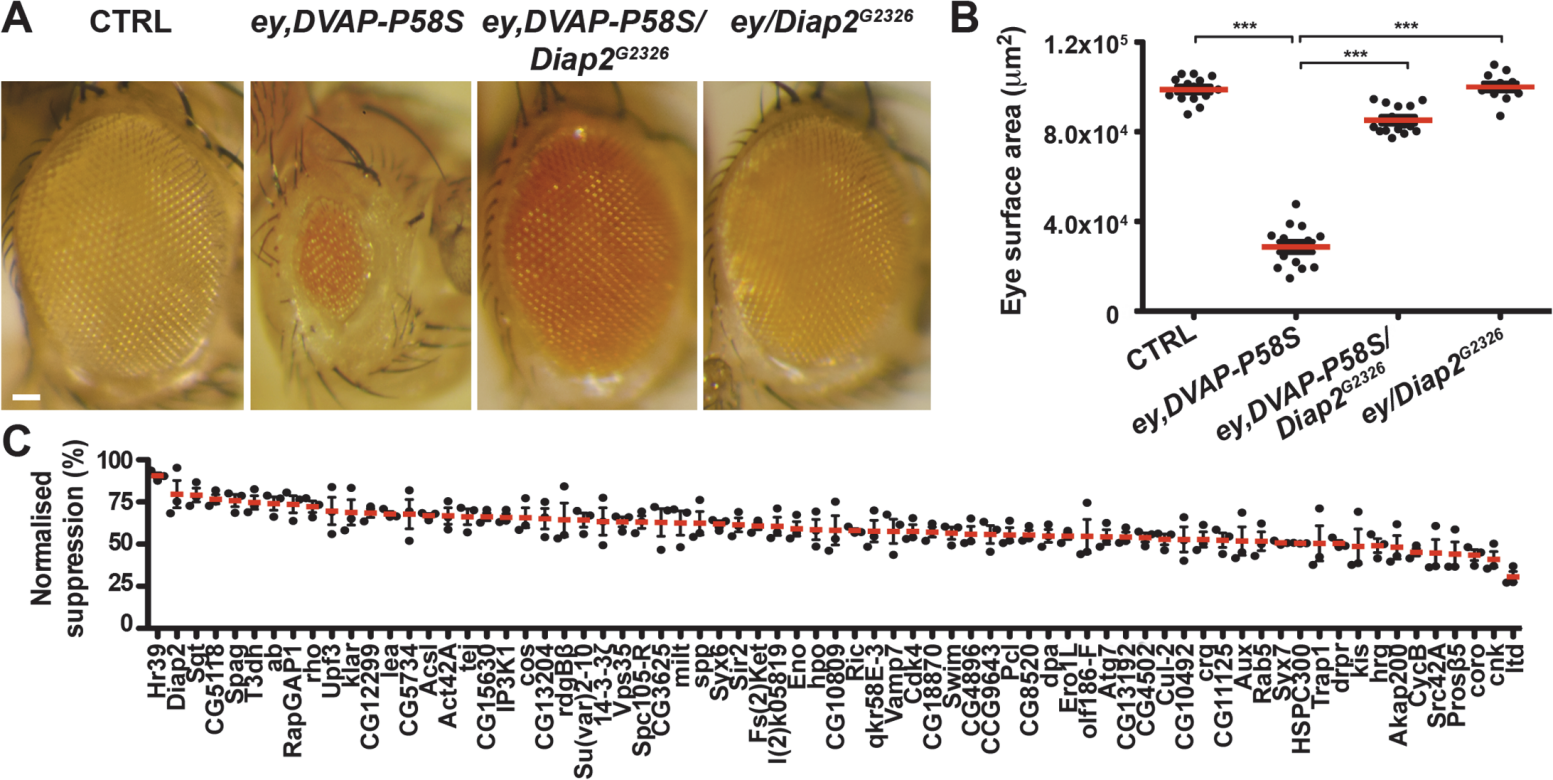
An overexpression screen for modifiers of the DVAP-P58S eye phenotype identified 71 suppressors. (A) Stereomicroscope images of external eyes from control flies (*ey-Gal4/+*), flies expressing *DVAP-P58S* in the eye (*ey*,*DVAP-P58S)*, flies expressing *DVAP-P58S* in the presence of an heterozygous overexpression allele of *Diap2* (*ey*,*DVAP-P58S/Diap2* ^*G2326*^) and flies overexpressing *Diap2* under the control of the *ey-Gal4* driver (*ey*,*Diap2* ^*G2326*^). In controls, eye morphology is made up of a regular array of ommatidia, however expression of *DVAP-P58S* induces a reduction in eye size and loss of ommatidial texture. Coexpression of *DVAP-P58S* and *Diap2* leads to a highly significant suppression of the eye phenotype while overexpression of the interacting gene on its own does not exhibit any phenotype. (B) Estimated eye surface area phenotype presented as scatter plots. Red horizontal lines represent the average surface area of the indicated genotypes. (C) Quantification of eye surface areas of modifiers with a suppression activity. The average eye surface area of each suppressor was measured, the area of the *ey-GAL4*,*DVAP-P58S* subtracted and the difference expressed as the percentage change from the eye size reduction associated with the ey,*DVAP-P58S* tester line. The strongest suppressor is expected to have a suppression value of 100% and an eye size similar to that of the wild-type. Conversely, a weak suppressor has a suppression value close to zero and an eye size similar to that of the *ey*,*DVAP-P58S* line. Results of three independent experiments for every suppressor are reported and red lines represent the average surface area for the indicated suppressors. ***P<0.001. n = at least 15 flies per genotype. Scale bar: 50μm.

**Fig 2:**
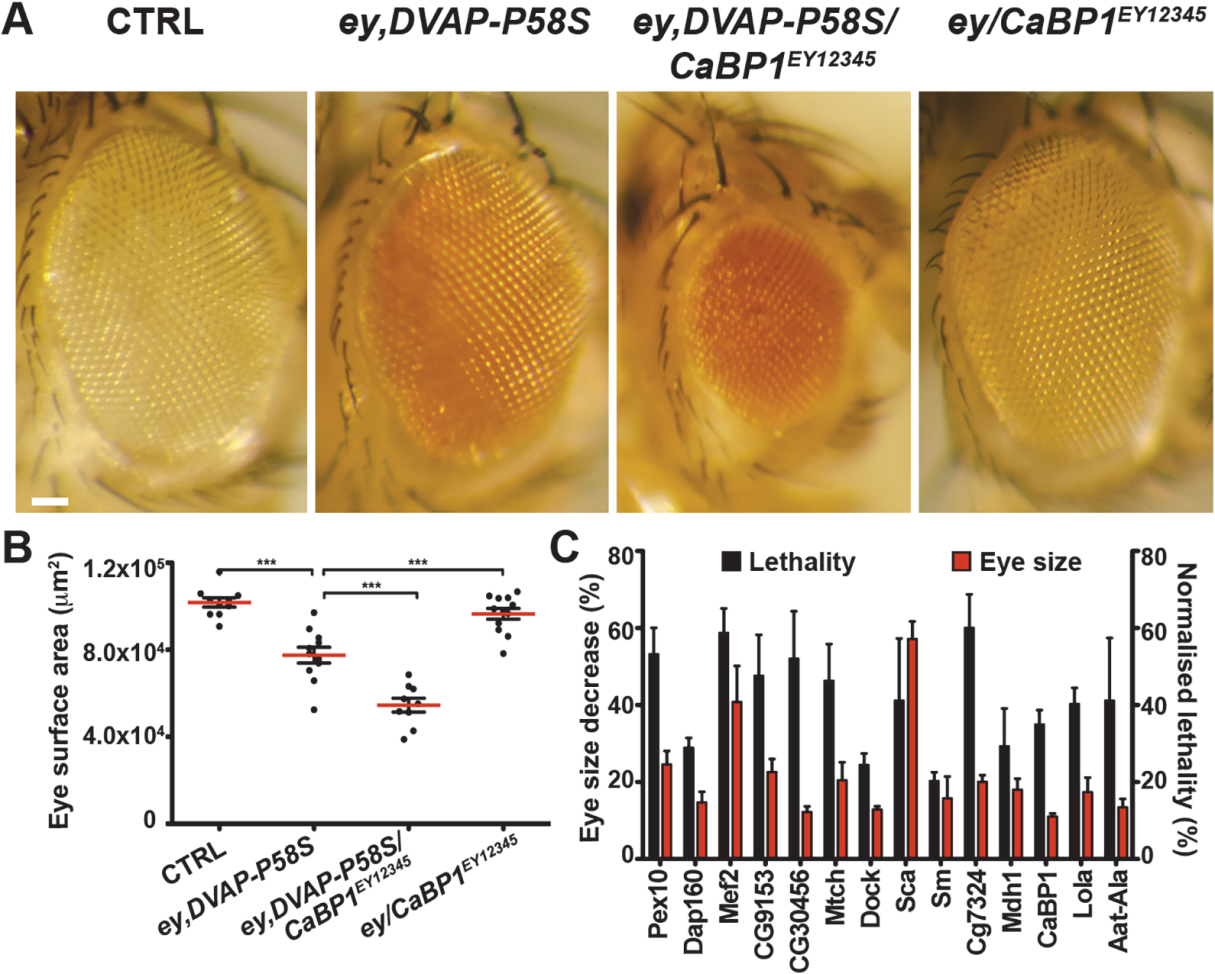
Enhancers of the DVAP-P58S eye phenotype. (A) Stereomicroscope images of adult fly eyes from controls (*ey-Gal4/+*), flies expressing *DVAP-P58S* in the eye (*ey*,*DVAP-P58S*), flies expressing *DVAP-P58S* in the presence of an heterozygous overexpression allele of the representative enhancer *CaBP1* ^*EY12345*^ (*ey*,*DVAP-P58S/CaBP1* ^*EY12345*^) and flies overexpressing *CaBP1* ^*EY12345*^ under the control of the *ey-Gal4* driver (*ey/CaBP1* ^*EY12345*^). (B) Estimated eye surface area phenotype presented as scatter plots. Red horizontal lines represent the average surface area of the indicated genotypes. (C) Quantification of the eye surface areas and lethality of modifiers with an enhancing activity. See Materials and Methods for the calculation of the lethality and the eye surface areas of every enhancer. Reported data result from at least three independent experiments. ***P<0.001. n = at least 15 flies per genotype. Scale bar: 50μm.

Fig. 1A depicts light micrographs of representative eye phenotypes of one suppressor and Fig. 1B shows scatter plots of actual surface areas for the same suppressor. Fig. 1C presents average surface areas of every suppressor resulting from a set of three independent experiments. In Fig. 2, results of a representative enhancer (Fig. 2A,B) and data on the effect of the remaining enhancers for viability and eye neurodegenerative phenotypes are presented (Fig. 2C). The complete list of all suppressors is reported in S1 Table together with the percentage of modifying activity of every modifier. S2 Table reports genes with an enhancing effect on both the eye and organismal lethality phenotypes.

In summary, this primary screen identified 85 modifiers including 71 suppressors and 14 enhancers(S2 Fig). The 7% hit rate for genetic modifiers among the 1,183 tested strains is double the number of 1–4% typically observed in similar genetic screens in *Drosophila*. This demonstrates that the adopted approach was successful in identifying genes interacting with the *DVAP-P58S* neurodegenerative phenotype. However, false positive are common in high- throughput screens and therefore the effect of these modifiers was carefully and quantitatively retested by using an independent allele and an additional assay.

### Genetic validation of the identified modifiers

Insertions of P-elements within or close to a gene could make difficult to assign phenotypes to specific genes with a high degree of confidence. However, if the ability of an EP or EPgy2 line to genetically modify the *ey-Gal4*,*DVAP-P58S* phenotype is due to a disruption of the gene associated with the insertion, then other mutant alleles for that gene should also show a modifying effect. On the basis of previous studies, we identified additional alleles for many modifiers and tested their ability to modify the phenotype associated with the *DVAP-P58S* construct.

For 62 out of the 85 modifiers, we were able to confirm the modifying effect of the specific gene on the *ey-Gal4*,*DVAP-P58S* phenotype using independently generated alleles obtained from other laboratories/stock Centers or by RNAi knock-down of the putative modifier(S3 Table, S2 Fig). More specifically, of the 71 suppressors 39 were confirmed by showing that an RNAi line has an enhancement effect on the eye/lethality phenotypes. Similarly, 3 suppressor lines were shown to be enhancers when loss-of-function alleles were tested to confirm their interacting ability. Conversely, the suppressor effect of 5 genes was confirmed by UAS lines while 3 suppressors were confirmed by using an independent EP or EPgy2 line. For 3 suppressors, the position and the orientation of the P-element suggest that the allele is likely to be a loss-of-function and therefore the suppression effect was confirmed by using an RNAi line (for *HSPC300* and *Hippo*) or an independent loss-of-function allele (for *Klarsicht*). Finally, the suppressive effect of the Syntaxin7/Avalanche gene was validated by using an antibody specific for the corresponding protein that was shown to be abnormally distributed in *ey-Gal4*,*DVAP-P58S* eye imaginal discs when compared to controls(S3 Fig). Of the 14 enhancers, 7 exhibited a suppressor effect with RNAi alleles and 2 were suppressors when loss-of-function mutations were tested. In summary, by using genetic and immune-histochemical approaches, the effect of 63 out of 85 modifiers was confirmed(S2 Fig).

For the remaining 22 lines (17 suppressors and 5 enhancers), it was not possible to confirm their effect by using RNAi lines. Although the relevance of these genes as potential modifiers of the DVAP-P58S phenotype needed to be confirmed by using additional independent alleles, for 21 of them the P-element insertion has been molecularly mapped and supposed to affect a specific gene. It is therefore conceivable that in these cases, RNAi alleles generated only a weak hypomorph so that the partial inactivation of the gene would not be sufficient to induce a visible effect. For the CG15630 suppressor line, however, the localization of the P-element insertion made it difficult to assign the modifying effect to a specific gene and therefore, at the moment, this gene remains a candidate modifier as it has been found to have an effect with only one allele.

In conclusion, a total of 63 genes out 85 were confirmed to be modifiers of DVAP-P58S-induced neurodegenerative phenotype in the adult eye since the modifying ability is caused by changes in activity/expression of interacting genes and not by unrelated or genetic background effects.

### The modifying effect of DVAP-P58S interacting genes is extended to the adult nervous system

**Fig 3:**
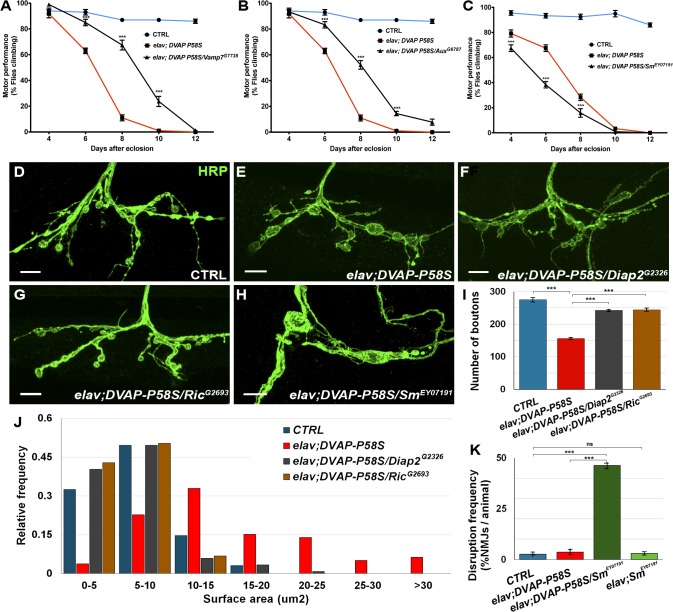
Effect of a subset of modifiers on motor and synaptic structure abnormalities associated with the expression of *DVAP-P58S* in neurons. (A, B) Representative analyses of two genetic modifiers showing suppression (*Vamp7* ^*G7738*^ in A and *Aux* ^*G6787*^ in B) and an enhancement (*Sm* ^*EY07191*^) in (C) of motor defects associated with pan-neural expression of DVAP-P58S (*elav;DVAP-P58S*). (D-H) Representative confocal images of NMJs stained with anti-HRP antibodies from *elav-Gal4/+* control (D) and *elav;DVAP-P58S* larvae (E). (F,G) NMJs expressing *DVAP-P58S* and either one of two suppressor genes, *Diap2* (*elav;DVAP-P58S/Diap2* ^*G2326*^ in F) or *Ric* (*elav;DVAP-P58S/Ric* ^*G2693*^ in G). (H) Representative images of NMJs stained with anti-HRP antibodies expressing *DVAP-P58S* and one enhancer, *Sm* (*elav;DVAP-P58S/Sm* ^*EY07191*^). (I) Quantification of total number of boutons on muscle 12 and 13 of abdominal segment 3 in *elav-Gal4/+* control (275.3 ± 6.0, n = 8), *elav;DVAP-P58S* (156.4 ± 2.9, n = 8), *elav;DVAP-P58S/Diap2* ^*G2326*^ (242.5 ± 3.3, n = 8) and *elav;DVAP-P58S/Ric* ^*G2693*^ (244.4 ± 4.1, n = 8) NMJs. (J) Quantification of bouton size at muscle 12 (type I and type III boutons) of abdominal segment 3 in *elav-Gal4/+* control, *elav;DVAP-P58S*, *elav;DVAP-P58S/Diap2* ^*G2326*^ and *elav;DVAP-P58S/Ric* ^*G2693*^ NMJs. The phenotypic decrease in number of boutons and increase in their size in *elav;DVAP-P58S* is strongly suppressed by the co-expression of either *Diap2* ^*G2326*^ or *Ric* ^*G2693*^ alleles (P < 0.001 in every case). (K) Disruption frequency of NMJs in *elav-Gal4/+* control (n = 94 NMJs/10 animals), *elav;DVAP-P58S* (n = 90 NMJs/10 animals), *elav;DVAP-P58S/Sm* ^*EY07191*^ (n = 87 NMJs/10 animals) and *elav;Sm* ^*EY07191*^ (n = 96 NMJs/10 animals) larvae. Quantification of disruption frequency is measured as the average percentage of NMJs with severe structural abnormalities. Synaptic architecture is rarely altered in controls and in neurons of *elav;Sm* ^*EY07191*^ larvae. While disruption is occasionally observed in larval neurons expressing *DVAP-P58S* compared to controls, this phenotype is significantly worsened by the co-expression of the enhancer *Sm* (P < 0.001). Bar = 10 μm. Error bars denote SEM. ***P < 0.001.

Our main goal with the primary screen was to generate a short list of candidates that could then be tested in a functional context that is more pertinent to the disease such as the motor system. We confirmed and extended our analysis by testing whether the identified modifiers also affect the DVAP-P58S-induced toxicity in neurons other than those of the adult eye and, more specifically, whether they interact with *DVAP-P58S* to affect motor behavior. Pan-neural expression of the *DVAP-P58S* construct under the control of the *elav-Gal4* line does not result in viable offspring at 30°C [15]. However, when flies of the same genotype are raised at 28°C, about 70% of the expected offspring is viable and surviving flies exhibit a progressive decline in locomotion ability. The motor performance of flies as a function of age was assessed and quantified using a climbing assay. Control flies show no significant decrease in their motor performance until later in life and, in particular, for the entire period during which the motor performance was tested, about 80% of control flies were still able to climb (Fig. 3A,B,C, blue lines). Conversely, flies expressing *DVAP-P58S* specifically in the nervous system using the *elav-Gal4* driver (*elav-Gal4;DVAP-P58S*), display progressive impairment of their motor performance (Fig. 3A,B,C, red lines). We then analyzed the effect of every modifier on the climbing phenotype associated with the expression of *DVAP-P58S* in neurons. As shown in Fig. 3A,B for representative examples of two suppressor genes (black lines), climbing ability was dramatically improved compared to flies that expressed only *DVAP-P58S*. Conversely, Fig. 3C (black line) reports an example of an enhancer where flies show a progressive worsening of the phenotype over the same time period.

Data for all modifiers are reported in S4, S5, S6, S7 Fig. Suppressors were classified as strong when they exhibited a consistent and robust suppression for at least three out of five time points tested(S4 Fig). Suppressors with an intermediate effect displayed a significant change in two time points out of five(S5 Fig). Finally, suppressors with a weak effect were those in which performance was improved over the entire time period but differences were of high statistical significance in only one time point or, mildly significant, over two time points (S6 Fig). Eight genes identified as enhancers in the primary screen also exhibit a strong enhancing effect on *DVAP-P58S*-induced motor dysfunction(S7 Fig). Out the 85 modifiers, 58 were found to have a modifying effect on DVAP-P58S motor performance(S2 Fig).

To investigate whether the locomotion defects could be attributed to synaptic deficits, we turned to the Neuromuscular Junction (NMJ) of third instar *Drosophila* larvae, a model glutamatergic synapse, the structural and functional properties of which have been extensively characterized [35]. We examined the morphology of larval NMJs located on muscle 12/13 by immunohistochemistry with antibodies against the presynaptic marker HRP. As previously reported, *elav-DVAP-P58S* NMJs exhibit a reduction in the number of boutons and an increase in their size when compared to controls [10,14]. Here we found that co-expression of either *Ric* or *Diap2*, two potent suppressors of the *DVAP-P58S*-dependent eye and locomotion phenotypes; also rescued the morphological defects of NMJs both in size and number of boutons (Fig. 3D,E,F,G, I,J). Notably, expression of either *Ric* or *Diap2* alone under the control of the same *elav-Gal4* driver does not result in any visible structural change at the synapse(S8 Fig). In contrast to what was observed for *Diap2* and *Ric*, co-expression of *Smooth* (*Sm*), a gene with an enhancing effect on the *DVAP-P58S*-mediated eye and motor phenotypes, leads to a severe disruption of synaptic structural integrity at NMJs. Specifically, while in controls synaptic boutons within a branch resemble a string of beads connected by a short neuritic process, the architecture of NMJs co-expressing DVAP-P58S and Sm is profoundly altered and consists of a reduced number of irregularly shaped boutons which are frequently disconnected from the nerve or axonal branches (Fig. 3D,H,K). Disruptions are very rare in controls and in *elav*,*Sm* synaptic terminals and are occasionally found in in *DVAP-P58S* expressing NMJs but their frequency is drastically increased by co-expression of *DVAP-P58S* and *Sm* (Fig. 3K). Finally, a small but statistically significant decrease in bouton number was observed when Smooth expression was induced by the driver alone(S8F Fig). However, this defect is very mild compared to the widespread disruption of synaptic structure due to the concomitant expression of DVAP-P58S and Sm.

In summary, the strong correlation between modifying effects on locomotion behavior and on synaptic morphology we reported for a subset of DVAP-P58S interacting genes, further supports the functional relevance of the identified interactions.

While most modifiers were isolated as suppressors and confirmed to have a suppressive effect in the motor assay, DAP160 was isolated as a suppressor in the eye but exhibited an enhancing effect on the motor phenotype when broadly overexpressed in neurons. This apparent contradiction suggests that perhaps a more complex interaction exists between *DVAP-P58S* on one hand and the *DAP160* gene on the other. Under these specific testing conditions, neuronal expression of the three modifiers (rhomboid, Src42 and leak) exhibit an independent developmental lethality thus impairing the testing of motor performance in adult flies. These genes and the remaining modifiers that were not confirmed by the motor assay still represented *bona-fide* interactors of *DVAP-P58S* phenotypes, although additional alleles or/and different assay conditions will be required to assess their effects on *DVAP-P58S* motor performance.

Importantly, these results show that our analysis identified 42 of the 85 tested as high-confidence genetic modifiers(S2 Fig, S6 Table). Indeed these genes exhibit a modifying effect on *DVAP-P58S* disease phenotypes by using more than one allele and two independent assays, the eye neurodegenerative phenotype and the adult fly motor performance. In conclusion, a large set of modifiers was found to affect both phenotypes, eye degeneration and locomotion ability, in the same direction and there is indeed a strong correlation between the severity of the modifying effect on eye neurodegeneration and that on locomotion impairment.

A reasonable test of success for any genetic screen would be the isolation of genes that we would predict to interact with the DVAP protein on the basis of prior experiments. Not all the *Drosophila* genes have been tagged with EP or EPgy2 insertions and we did not screen all the available lines in the collections. Therefore the isolation of even a few such modifiers would confirm that the screen has been successful. Indeed, we isolated several such genes. Most notable among them is rdgBβ. The gene rdgBβ is predicted to encode a phosphoinositide phosphatase that has not been extensively investigated hitherto. Previous studies suggest that rdgBβ may have functions similar to those of the better characterized phosphodiesterase rdgB [36]. rdgB is the homologue of the human Nir2 gene that has been shown to interact with the hVAPB protein to control Golgi transport in human cell lines [37]. In addition, we and others have shown that a primary and conserved function of VAPB proteins is to control the levels of phosphoinositides associated with cellular membranes [15,38,39]. Another gene isolated as a strong modifier is leak. In *C*.*elegans*, the N-terminal MSP domain of VAPB is cleaved and secreted, serving as an extracellular ligand. The secreted MSP fragment binds LAR and Robo family receptors on the muscle cell surface leading to alterations in actin organization and changes in mitochondrial morphology and function [7,8]. Leak is the *Drosophila* homologue of Robo2, a protein family controlling axonal pathfinding and tracheal system development [40]. In *DVAP* mutants, muscle phenotypes similar to those associated with the removal of the corresponding gene in *C*.*elegans* have been reported [7,8]. It is therefore possible that if leak is a Robo2 receptor, the similarity in phenotypes between *Drosophila* and nematodes corresponds to a similarity in molecular mechanisms underlying VAPB-mediated functions. In addition, among the high-confidence *DVAP-P58S* interacting genes, our screen identified actin (S1 Table) and mutations in profilin 1, which regulates actin polymerization, were recently linked to ALS [41,42]. Our studies also uncover modifiers for which no involvement in VAPB function has been previously reported. Analysis of these genes could potentially illuminate novel and so far unpredicted, functional processes involved in ALS8 pathogenesis.

### Computational network analysis demonstrates that multiple processes are involved in *DVAP-P58S*-mediated toxicity

Although we analyzed a large collection of genes, our screen was not saturating as less than 10% of the about 12,000 *Drosophila* genes were screened. Additionally, important modifiers may have induced subtle changes in DVAP-P58S pathology, which would have been excluded based on our strict criterion of robust suppression or enhancement of *DVAP-P58S*-associated phenotypes. Therefore, we performed an extensive computational analysis of modifier genes in order to extrapolate cellular processes, pathways and other genes that may have a relevant role in DVAP-P58S-associated disease phenotypes. In the list of modifiers we also incorporated DVAP as it has been shown that the wild-type protein binds to the ALS8-causing allele and is sequestered into the aggregates. In addition, it appears that at least some of the interactions of VAPB proteins are retained by the mutant allele [15,16]. It is therefore possible that the wild-type version of the protein has an important role to play in the disease pathogenesis.

**Fig 4:**
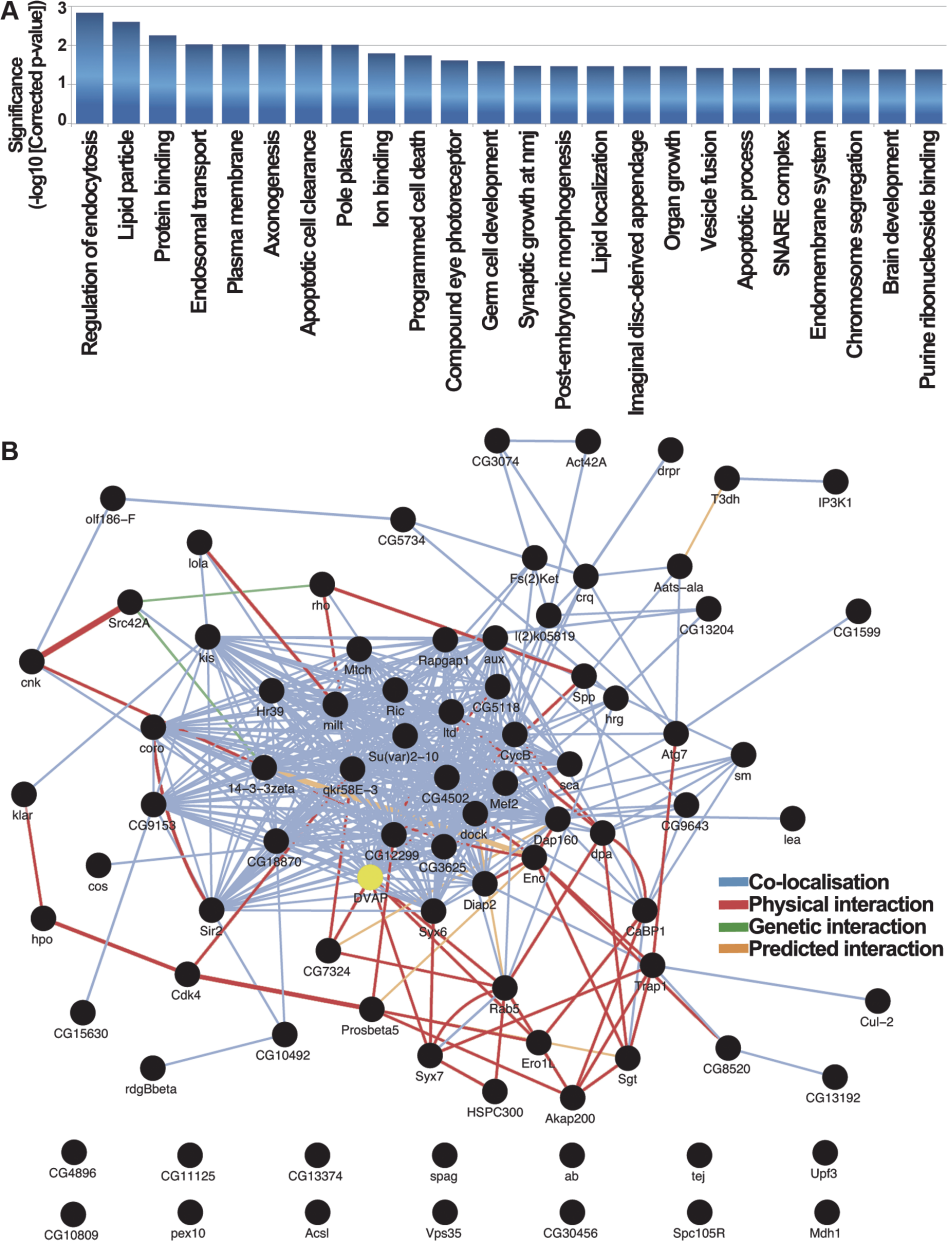
Functional role and interactome analysis of genetic modifiers in *Drosophila*. (A) *Drosophila* functional categories overrepresented in the genetic modifiers list. Enrichment significance is expressed as the-log10 transformed Benjamini-Yekutieli corrected p-value resulting from hypergeometric tests of GO-term enrichment. In this process we de-correlate the modifier gene list annotated GO-tree to remove GO-term conditional dependence using the weighted elimination method [43] so that we can perform multiple testing correction. (B) *Drosophila* interaction network for *DVAP-P58S* screen hits identifies connections between most of them. 72 of the 85 genes that modify the *DVAP-P58S* phenotypes are annotated as black circles while network edges are colored accordingly to the type of interaction. 13 of the 85 genes are excluded from the interactome that includes also DVAP (yellow).

The 85 modifier genes were analyzed using the R/Bioconductor package ‘topGO’ which uses a GO-graph de-correlation method to remove conditional dependence from the annotated GO tree improving interpretation of results by removal of non-specific higher level terms and allowing for multiple testing correction to control the false positive rate [43]. The predominant processes enriched in the modifier list reflect broad effects on a variety of biological processes reinforcing the concept of the pleiotropic nature of disease pathogenesis (Fig. 4A). Analysis of the *Drosophila* DVAP-P58S network with topGO identified endosomal regulation, lipid particle metabolism, vesicular trafficking and apoptosis as enriched functional categories (Fig. 4A). Some of these biological processes, such as vesicular trafficking, have already been linked to VAPB activity although their role in VAPB-mediated neurodegeneration has never been directly assessed [44,45]. Additionally, functional categories such as endocytosis and lipid particle metabolism represent biological processes that have not been previously associated with VAPB activity (Fig. 4A). Based on a recent report showing that VAPB can function as an oncoprotein in humans [46], it is particularly intriguing that a number of modifiers fall within enriched functional categories related to apoptosis (Fig. 4A).

To further extend our analysis, we searched the GeneMania database [47] to determine whether the genetic modifiers are interconnected through physical and genetic interactions as well as colocalization studies. We found that 72 of the 85 genes are linked into a complex network and, more importantly, 7 of the *DVAP-P58S* genetic modifiers (CG7324, Syx7, Ero1L, Rab5, CG5118, rho and Spp) have previously been reported to be physical interactors of DVAP [48] (Fig. 4B).

We also carried out a statistical analysis comparing our GeneMania derived network graph to those produced by repeatedly sampling the GeneMania data set with 1000 randomised gene lists of the same size. To summarise the topographical properties of the resulting groups of network graphs we calculated the mean node-degree and mean node-betweeness, two properties for which biological networks have been shown to have high values [49] and calculated Z-scores for the comparison of both metrics between our graph and the distribution of random gene graphs. We found significantly higher values for both mean node-degree (2.07x, Z = 4.67, p = 3.05x10^-6^) and mean node-betweeness (2.86x, Z = 2.75, p = 0.006) consistent with our interpretation that the network of DVAP-P58S modifiers is structurally very different from that produced from random gene lists and shares key topographical features commonly observed in other known biological networks.

Together, these findings confirm and expand categories and pathways associated with DVAP function and provide us with a perspective on the diverse molecular functions that can modulate DVAP-induced pathogenesis in vivo.

### Building the human interactome of DVAP-P58S genetic modifiers

Careful *Drosophila* to human homology mapping using a recently developed orthologue mapping tool (*Drosophila* RNAi Screening Center Integrative Orthologue Prediction Tool, DIOPT at http://www.flyrnai.org/diopt) shows that of the 85 *Drosophila* genes at least 77 have human orthologues (S4 Table). We decided to use DIOPT instead of any other specific software because it has the advantage of integrating the results of several orthologue searching tools based on different algorithms [50]. This tool also includes algorithms identifying potential functionally related proteins in humans based on information derived from protein-protein interaction networks. In searching for orthologues using this tool, we applied a filter that removed predicted human proteins with a DIOPT score less than 2 as this would mean that the identification of this orthologue is supported by only one algorithm.

To search whether any of the identified modifiers are known to be associated with a disease of the nervous system, we used a tool based on DIOPT named DIOPT-DIST (http://www.flyrnai.org/diopt-dist). The DIOPT-DIST website links *Drosophila* genes to high-confidence orthologues of disease genes extracting information from the Online Mendelian Inheritance in Man data set and Genome-Wide Association Studies. We found that a considerable number of human orthologues of genetic modifiers have been associated to neurological disorders including neurodegenerative diseases such as Parkinson’s and Alzheimer’s diseases, multiple sclerosis and spinocerebellar ataxias. Other genes were linked to human psychiatric disorders such as schizophrenia, autism spectrum disorder and mental retardation(S5 Table). The recovery of these genes suggests that the genetic network identified by our screen may overlap, perhaps significantly, with the genetic networks associated with other human neurological disorders. If this turns out to be true, the use of *Drosophila* to explore other neurological disease networks via genetic screens would have the potential to identify common therapeutic targets that could be tested in other disease models.

**Fig 5:**
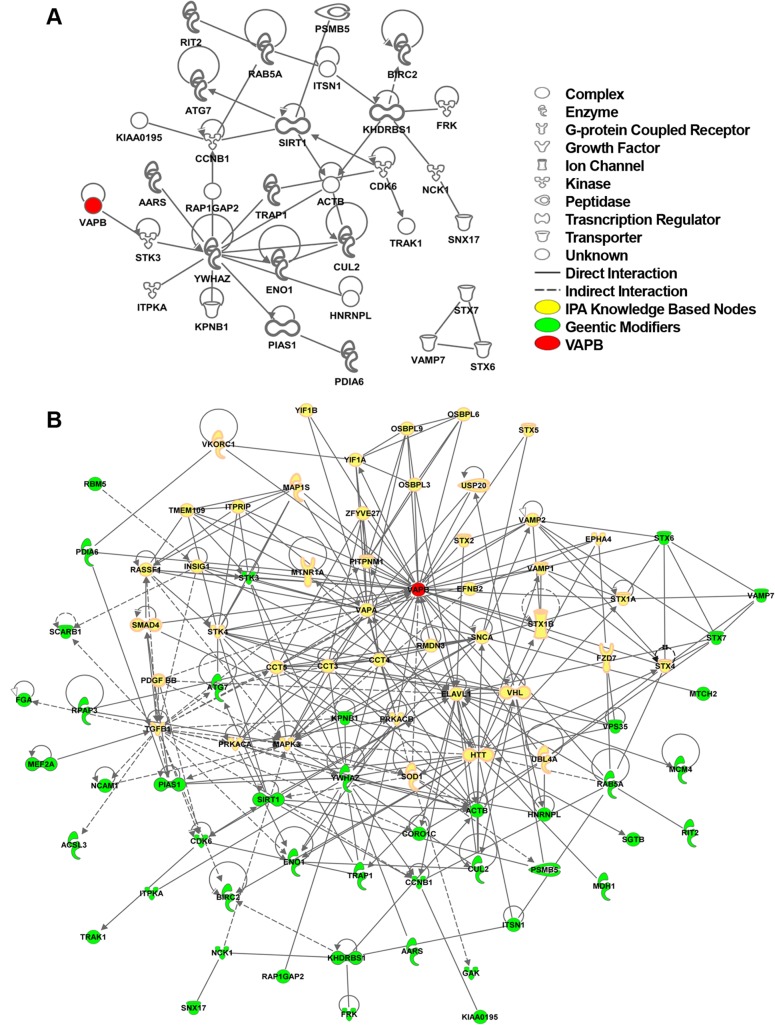
hVAPB human interactome. (A) IPA network between human orthologues of the *Drosophila* genetic modifiers that can be connected to each other without intervening nodes. IPA analysis indicates that about 40% (28 genes out of 77) of the human orthologues of *DVAP-P58S* genetic modifiers are connected in a network with other modifiers and hVAPB (red). In addition, three modifiers (STX7, VAMP7 and STX6) have interactions between them but not with hVAPB interactome. Nearly 60% of the modifiers (46 genes) have no interactions that would connect them to hVAPB or other modifiers. Icons represent the function of the node accordingly to the legend. (B) Extended human genetic network of hVAPB. Adding the direct and indirect interactors of hVAPB to the network shown in (A) generated a novel and extended network. In this interactome, a total of 44 modifiers (green) are connected to hVAPB through 43 additional proteins from the IPA database (yellow). Direct interaction (solid line) indicates direct physical contact between two molecules, e.g. binding or phosphorylation. Indirect interaction (dashed lines) indicates a functional interaction that does not require a direct contact between the two molecules (*e*.*g*. signaling events).

To study the *DVAP-P58S* genetic circuitry in the human context we generated a human view of the genetic *Drosophila* interactome taking advantage of the manually curated source of human molecular interactions from the Ingenuity Pathway Analysis (IPA database, Ingenuity system at www.ingenuity.com). This database integrates human gene relationships derived from a variety of experimental approaches including proteomic studies. Using the hVAPB proteins and the 77 human genes homologous to the genetic modifiers identified in *Drosophila*, we derived the human interaction network of hVAPB. The generated human interactome includes hVAPB and 31 additional genes indicating that these proteins, which represent 40% of the identified modifiers, are interconnected in the human IPA database (Fig. 5A). Importantly, we found that all the genes included in the hVAPB interactome are also components of the *Drosophila* genetic network further supporting the relevance of the fly modifiers for the elucidation of the function of hVAPB and for ALS8 pathogenesis.

To further expand the hVAPB interactome, we identified proteins in the IPA database that interact with hVAPB and incorporated them into the list of human orthologues of *Drosophila* modifiers. In so doing, we established a new network of interconnected genes that include the initial 31 modifiers and 12 additional ones (Fig. 5B). Our experimental data integrated with our computational analysis support and reinforce the relevance of the *Drosophila* model in identifying the cellular processes and in dissecting the complex genetic network underlying ALS8 pathogenesis in humans.

### Computational and experimental analysis identifies endocytosis as a process implicated in ALS8 pathogenesis

**Fig 6:**
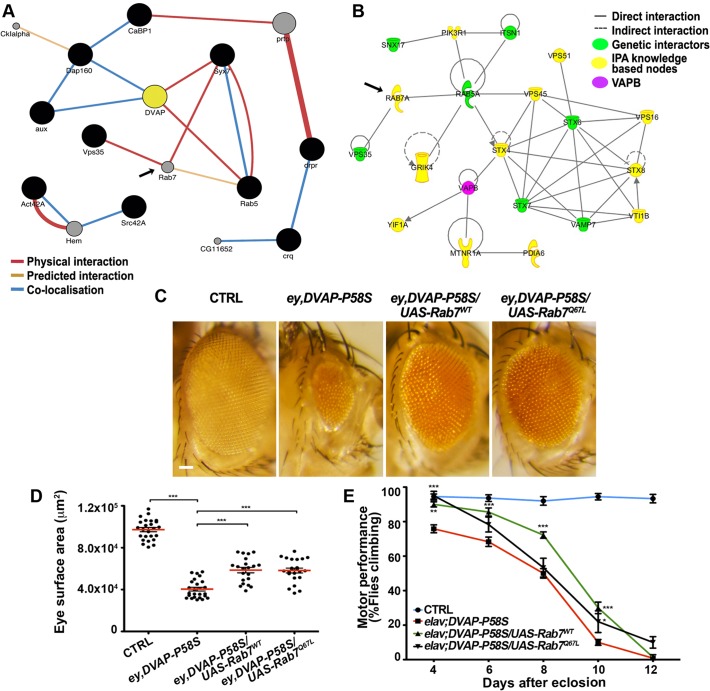
Computational and experimental analyses identify Rab7 as a modifier of the DVAP-P58S-mediated phenotypes. (A) Sub-network interactome of *Drosophila* modifiers (black circles) involved in endocytosis and vesicular trafficking. Predicted intervening nodes are depicted in grey. Color of edges indicates the type of interaction according to the legend. (B) Interactome of human orthologues of *Drosophila* modifiers involved in endocytic and vesicular trafficking in green with intervening nodes predicted by the IPA system in yellow. Rab7 is indicated as predicted interacting gene in both networks. (C) Stereomicroscope images of external eyes from control flies (*ey-Gal4/+*), flies expressing *DVAP*-P58S in the eye (*ey*,*DVAP- P58S*), flies expressing *DVAP-P58S* in the presence of either a wild-type (*Rab7* ^*wt*^) or constitutively active (*Rab7* ^*Q67L*^) allele of Rab7. Quantification of the suppression effect on the eye neurodegenerative phenotype (D) and motor performance (E) for flies of the indicated genotypes. Scale bar: 50μm.

To perform a further functional characterization of a subset of modifiers, we focused on genes that were present within both the *Drosophila* modifier network and the expanded IPA human network of hVAPB. A number of these genes include genes known to function in vesicular and endocytic trafficking. Then we generated a sub-network including protein interactions relevant to DVAP-P58S and proteins with Gene Ontology annotations related to vesicular trafficking and/or endocytosis present in the GeneMania data base (Fig. 6A). A similar network was defined for the human orthologues of these genes using IPA (Fig. 6B). Rab7 is among the genes that are predicted to be part of both the fly and human network but that were not identified in the screen. Rab7 is an endocytic marker of late endosomes that results from the maturation of early endosomes labeled by Rab5. Rab5 is a potent modifier of DVAP-P58S phenotypes and we showed that up-regulation of Rab5 functions as a potent suppressor(S1 Table).

To assess whether a similar effect could also be extended to Rab7, we crossed *ey-Gal4*,*DVAP-P58S* flies with flies expressing either a wild-type or a constitutive active form of Rab7 and, in both cases, a strong suppression of the disease phenotypes was observed. (Fig. 6C,D,E). While Rab5 and Rab7 provide important organelle identity markers for early and late endosomes respectively, Rab11 is instead the identity marker for recycling endosomes. As for Rab5 and Rab7, overexpression of a wild-type version of Rab11 suppresses the DVAP-P58S eye neurodegenerative phenotype(S9 Fig). These results are particularly striking if we consider that VAPB has been shown to bind to Rab7 and to be required for the proper spreading of late endosomes throughout the cell [51,52].

**Fig 7:**
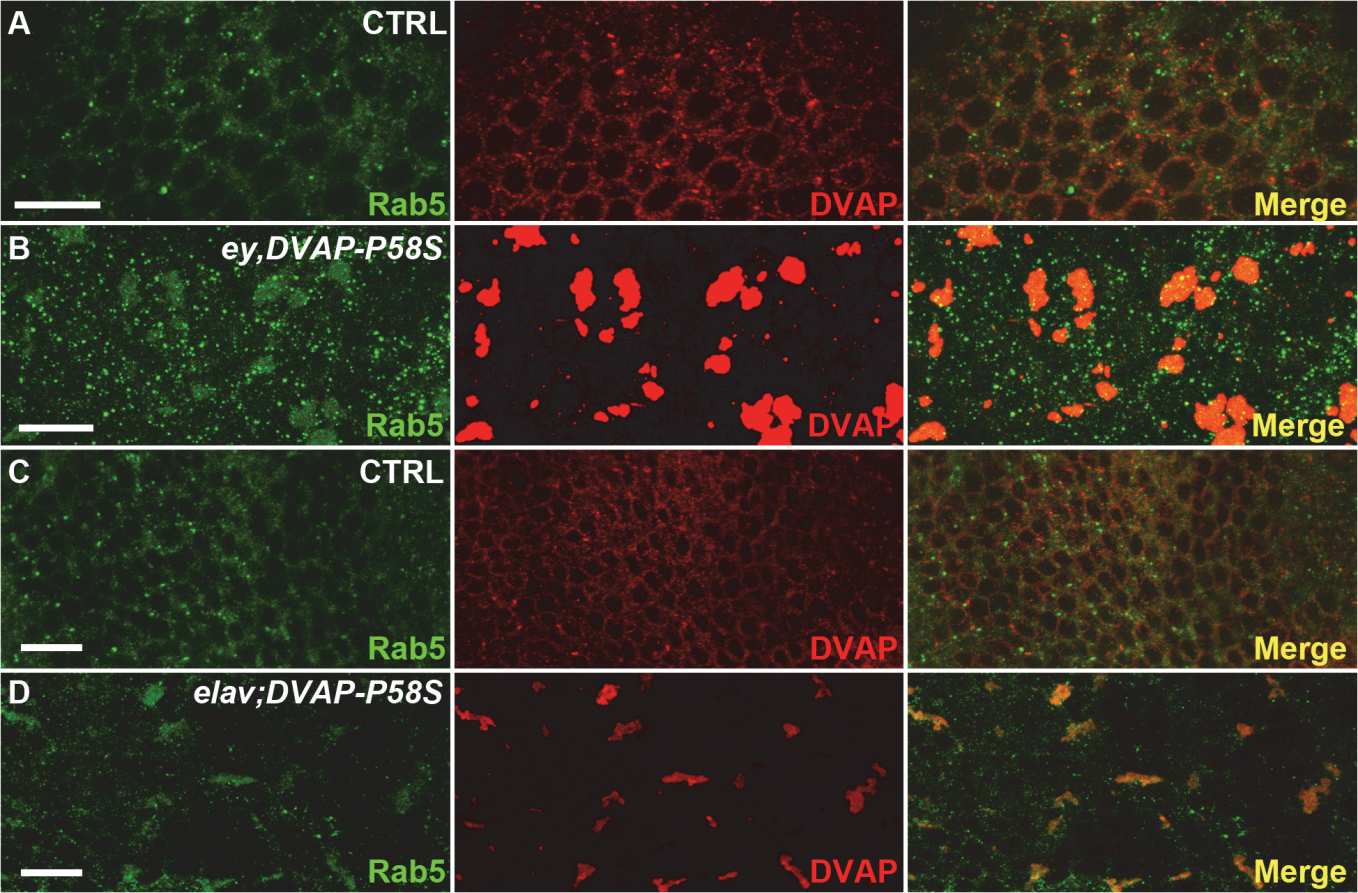
Abnormal accumulation of Rab5 in larval eye imaginal discs and brains expressing *DVAP-P58S*. (A) control *ey-Gal4/+* eye imaginal discs and *elav-Gal4/+* brains (C) immuno-stained with anti-DVAP and anti-Rab5 antibodies. Eye imaginal discs (*ey*,*DVAP-P58S* in B) and larval brains (*elav;DVAP-P58S* in D) expressing the pathogenic allele were stained with the same antibodies. In controls, DVAP and Rab5 are homogenously distributed throughout the cytoplasm but form large, overlapping aggregates in DVAP-P58S expressing cells. Scale bars: 10μm.

To test the cellular distribution of endosomes, eye imaginal discs expressing the DVAP-P58S transgene were stained with an antibody specific for Rab5. While in controls Rab5-positive immuno-reactivity forms a granular pattern dispersed throughout the cell, Rab5 abnormally accumulates in cells expressing the *DVAP-P58S* transgene and partially overlap with DVAP-P58S-induced aggregates (Fig. 7A,B). A similar effect was observed in neurons of larval brains expressing DVAP-P58S under the control of the pan-neural driver *elav-Gal4* (Fig. 7 C,D). These data suggest that inclusion of Rab5 proteins into aggregates and their inadequate delivery to the normal site of function may underlie DVAP-P58S-mediated neurodegeneration. Interestingly, we found that in eye imaginal discs simultaneously expressing Rab5 and DVAP-P58S, Rab5 immunoreactivity exhibits, at least in part, a normal distribution(S10 Fig). These data suggest that suppression of DVAP-P58S toxicity by overexpression of Rab5 proteins occurs by replacement of those proteins that have been incapacitated by inclusion into DVAP-P58S-induced aggregates. However, the elucidation of the mechanism whereby Rab5 protein expression confers protection against DVAP-P58S-associated neurodegeneration, requires further investigation.

Collectively computational and experimental evidence provide proof that our data set is enriched for proteins such those involved in vesicular trafficking and endocytosis and that these processes are important in DVAP function and patho-biology.

### Rab5 accumulates abnormally in motor neurons of patients affected by ALS

**Fig 8:**
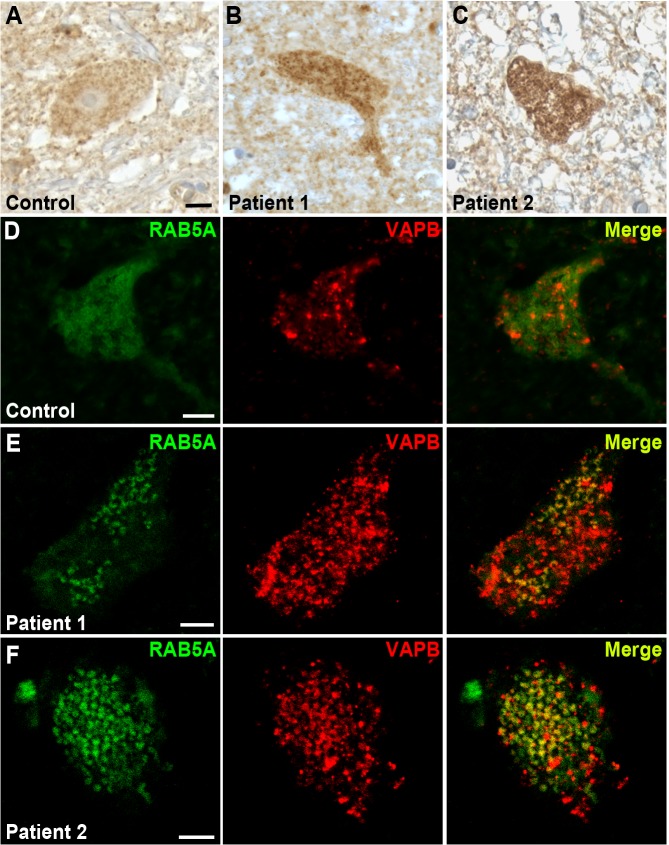
RAB5 accumulates in the cytoplasm of spinal cord neurons of patients with ALS. (A-C) Immuno-enzymatic detection of Rab5 in *post-mortem* tissues from ALS patients (B,C) and controls (A). RAB5 staining shows punctuate and diffuse distribution of immunoreactivity throughout the cytoplasm, whereas RAB5 accumulates in cytoplasmic clusters of spinal cord neurons of patients affected by ALS. (D-F) Immunofluorescence staining with anti-RAB5 and anti-hVAPB antibodies of spinal cord neurons from controls (D) and ALS patients (E and F). In ALS tissues, RAB5 redistributes to form clusters that overlap with hVAPB-positive immunoreactivity. Scale bar: 10μm.

We next performed immunohistochemistry on human *post-mortem* spinal cord tissue to determine whether RAB5 is present in motor neurons and whether its localization is affected in ALS. RAB5 is robustly expressed in spinal cord motor neurons and localizes to punctuate, granular pattern distributed throughout the cellular cytoplasm (Fig. 8A). Conversely, RAB5 localization in autopsy tissues from two sporadic ALS cases reveals an abnormal accumulation and clustering of RAB5 positive vesicles (Fig. 8B,C).

To analyze whether there was a co-localization between hVAPB and RAB5, we immunostained *post-mortem* cervical sections of spinal cord from ALS patients and age-matched controls. In controls, hVAPB was homogenously distributed throughout the neuronal cytoplasm and showed a granular appearance consistent with the protein being localized in the endoplasmic reticulum (Fig. 8D). In agreement with what previously described [17,18], we also noticed hVAPB expression was abundant in large motor neurons characterized by a prominent nucleus and nucleolus while smaller cells were still positive for Rab5 immunoreactivity but essentially devoid of hVAPB staining. In ALS tissues however, hVAPB staining was more conspicuous than in controls and present as punctuate accumulations that partially overlap with redistributed Rab5 positive clusters (Fig. 8E,F). Previous studies have reported that in a few ALS cases the expression levels of hVAPB protein is decreased. Reasons that could explain this apparent discrepancy are not known but they could include differences in disease characteristics such as disease duration and age of onset. Interestingly, we reported that in *Drosophila* expression of DVAP wild-type protein is sufficient to trigger many hallmarks of the disease and that a recently identified ALS causing mutation in hVAPB mirrors the effects of DVAP wild-type overexpression and acts as an allele with increased wild-type activity [11]. These data indicate that in humans as well as in fly models both gain and loss of function mechanisms may play an important role in hVAPB-induced ALS.

Notably, a recent study has also reported an abnormal accumulation of RAB5, RAB7 and RAB11 in *post-mortem* tissues of ALS patients [53]. Taken together, these data further support the notion that endocytic trafficking is important for ALS8 pathogenesis and that aberrant accumulation of endocytic markers can contribute to the human pathology.

## Discussion

A mutation in the hVAPB gene was initially reported to be causative of a range of motor neuron diseases including ALS8 [3]. The disease due to the P56S mutation in a conserved domain of the protein is characterized by a high degree of heterogeneity in age of onset, severity and clinical progression [3,54,55]. In a fly model of ALS8, severity of disease phenotypes has been reported to strongly depend on the dosage/activity of the mutant allele suggesting that genetic changes that either directly or indirectly affect DVAP function may be of therapeutic interest [9,15]. Though VAPB proteins have been linked to a number of seemingly unrelated functions [6,12,15,16,56], their interacting genes have not been systematically investigated nor has the link between ALS8-causing mutations in the hVAPB gene and the specific loss of motor neurons been uncovered. To systematically explore the genome for genes that are capable of modulating hVAPB-mediated disease phenotypes in vivo, we took advantage of the ALS8 fly model we recently developed [15]. This model offers the possibility of performing large-scale genetic screens by using two phenotypic readouts: the neurodegenerative phenotype and the motor behavior analysis associated with expression of the disease-causing allele in the eye and the nervous system, respectively [15, this study]. We identified numerous *DVAP-P58S* genetic modifiers that fall within a variety of different functional categories suggesting a pleiotropic genetic effect of ALS8 mutations in inducing neuronal dysfunction and death.

Overwhelming evidence supports a role of protein degradation deficits in neurodegenerative diseases including ALS through disruption of either of the two major protein clearance pathways: the ubiquitin proteasome system and autophagy [57]. Autophagosomes accumulate in the spinal cord of sporadic ALS patients [58] and a decrease in autophagic flux is present in ALS mice and cell lines expressing mutant SOD1 [59]. Here we report that the autophagy-related 7 (Atg7) protein, essential for autophagy, is a high-confidence suppressor of *DVAP-P58S* as overexpression of Atg7 suppresses and its down-regulation enhances the *DVAP-P58S*-associated disease phenotypes(S6 Table). These data are consistent with a scenario in which the autophagy process is down-regulated in *DVAP-P58S* expressing cells and are in agreement with previous experiments reporting that mice lacking Atg5 and Atg7 in the nervous system exhibit neurodegeneration [60,61].

The identification of ALS-causing mutations in genes affecting the ubiquitin-proteosome system directly supports a role for this process in ALS pathogenesis. These genes include ubiquilin-2, p62/SQSTM1, optineurin, vasolin-containing protein, and charged multivesicular body protein 2B ([2] and references therein). The ubiquitin-mediated protein degradation pathway ensures that only proteins that are properly folded and assembled are transported to their final destination. When mis/unfolded proteins are not degraded, an ER stress response is induced, known as unfolded protein response (UPR). Previous studies have already implicated VAPB proteins in protein homeostasis by showing that they can modulate the activities of the IRE1 and ATF6 arms of the UPR [4,62].

Here, we report that several DVAP-P58S interacting genes including Cullin2, CG9153 and CG4502, are involved in ubiquitin-mediated proteolysis. The ubiquitin-proteasome system is a selective protein degradation pathway in which a substrate is first tagged with a chain of ubiquitin and the resulting modified protein is then recognized by the proteasome where its proteolysis takes place. The process of ubiquitination involves a three branched enzymatic cascade. First, the chemically inert ubiquitin molecule is activated in an ATP-dependent reaction by binding to an E1 activating enzyme. Second, ubiquitin is transferred to the active site of an E2 ubiquitin conjugating enzyme. In the final step an E3 ubiquitin ligase functions to orchestrate the transfer of ubiquitin to a substrate protein that needs to be tagged for degradation [63]. Cullins organize the largest class of RING-E3 ubiquitin ligases by functioning as molecular scaffolds tethering a substrate target unit with the ring finger component that recruits the E2 ubiquitin conjugating enzyme [64]. Another type of E3 ubiquitin ligases is represented by the Hect type. The single molecule HECT-type E3 ligases are characterized by a Homologous to the E6-AP carboxyl terminus (HECT) domain that forms a thio-ester intermediate with ubiquitin as a pre-requisite for ubiquitin transfer to the substrate protein [65]. The CG9153 *DVAP-P58S* interacting gene encodes a putative HERC3 homologue in *Drosophila*. HERC3 contains a HECT domain and has been reported to associate with ubiquilin-2, an ALS causative gene [66]. Finally, another modifier is the CG4502 that encodes a predicted E2 ubiquitin ligase. Collectively these data provide further support to the notion that the ubiquitin-mediated protein clearance system represents a major component of ALS pathogenesis.

However, we were surprised to find that our list of modifiers is highly enriched for proteins linked to lipid droplets (LDs), by either functional or proteomic studies. As an example, the Acyl-CoA synthetase long-chain (*Acsl*) gene is a high-confidence modifier of *DVAP-P58S* phenotypes (S6 Table) and its vertebrate homologue ACSL3 has been implicated in LD biogenesis [67]. LDs are ubiquitous organelles that collect, store and supply lipids [67]. The absence of ACSL3 significantly reduces nucleation of emerging LDs, impairs accumulation of neutral lipids and decreases size and numbers of mature LDs [67,68]. Here we show that overexpression of *Acsl* ameliorates, while its down-regulation exacerbates, both neurodegeneration and motor disabilities associated with neuronal expression of DVAP-P58S. These data indicate that *Acsl* could be down-regulated in *DVAP-P58S* neurons and that impaired LD biogenesis may represent an important pathological aspect of VAPB-mediated ALS.

Importantly, we also found that loss of function mutations in the *Drosophila* gene *Klarsicht* controlling motion and distribution of LDs [69] acts as a potent suppressor of DVAP-P58S phenotypes (S6 Table). Aberrant movement of droplets can compromise their dispersion throughout the cytoplasm. In *C*.*elegans* and mice, mutations in VAPB induce an aberrant clustering of LDs in striated muscles that causes an energetic unbalance and a decrease in ATP production following starvation [70]. Consistent with these data, large-scale proteomic studies showed that DVAP is an LD-associated protein [71].

The functional role of LDs in the nervous system has been largely neglected, though recent studies have reported that a number of LD proteins are abundantly expressed in the brain [72,73] and some of them have been directly linked to motor neuron diseases. One such protein is seipin. Gain-of-function mutations of seipin cause motor neuron diseases known as Silver syndrome/spastic paraplegia 17 and distal hereditary motor neuropathy type V [74]. Seipin is an ER resident protein that regulates LD morphology in yeast while in mammals is essential for the induction of lipogenesis [75–77]. Mice expressing the seipin transgene carrying the disease-causing mutations, develop symptoms of motor neuron diseases along with an up-regulation of ER stress markers and an induction of autophagy [78]. In addition, spartin/SPG20, a protein mutated in the motor neuron disease known as the Troyer’s syndrome, is found on LDs and is implicated in LD turnover [79]. Interestingly, it has been proposed that LDs may play a protective role as they are large hydrophobic surfaces that sequester un-/mis-folded proteins and prevent their accumulation in large inclusions [80]. This protective function may be of general relevance also for motor neuron diseases for which the link with LD metabolism is not directly evident. Indeed, SOD1 mouse models of ALS have been shown to benefit from a fat rich diet that appears to attenuate disease symptoms [81]. Taken together, these data suggest the intriguing possibility that maintaining normal levels of neutral lipid content may represent a novel and promising therapeutic strategy in ALS.

Here we show that computational and experimental analyses support a role for the endocytic pathway in ALS8 pathogenesis. The endosomal system is necessary for regulating, sorting and degrading proteins via autophagy or the ubiquitin proteasome-system. Rab-GTPases are identity markers of endosomes and regulate endocytosis through interactions with vesicular coat components, motor proteins and SNARE proteins. Incoming substances and receptors for the regulation and fine-tuning of many cellular pathways are initially present on Rab5-containing early endosomes that undergo maturation to become Rab7-containing late endosomes while Rab11 regulates recycling of endocytosed proteins via recycling endosomes [82]. Overexpression of several Rab-GTPases proteins including Rab5, Rab7 and Rab11, functions as potent suppressor of DVAP-P58S associated phenotypes. Further supporting a link between hVAPB and endocytosis, we identified as modifiers the SNARE proteins VAMP7 and Syntaxin 7, which are required for late endosome-lysosome fusion events [83]. We also isolated as a modifier the VPS35 gene, coding for a component of a retromer complex, which guides protein sorting from the endosomal-lysosomal degradation pathway retrogradely to the Golgi network [84]. Furthermore, we found an abnormal distribution of RAB5 into clusters overlapping with hVAPB accumulations in ALS patients, suggesting hVAPB-mediated dysregulation of endosomal trafficking in ALS pathogenesis. These data were confirmed by a recent report showing aberrant accumulation of RAB7- and RAB11-positive vesicles in *post-mortem* tissues of ALS patients [53]. Interestingly, previous studies have shown that DVAP binds Rab7 and controls intracellular positioning and distribution of late endosomes [51,52]. These data collectively indicate that VAPB proteins have a broad and conserved function in facilitating endocytic trafficking and that disruption of this process is a prominent cause of ALS pathogenesis.

Among the genes our genetic strategy identified as DVAP-P58S modifiers, there are several members of the Ras signaling pathway, indicating that disruption of this pathway may play a role in ALS pathogenesis. The Ras pathway, which enables cells to respond to external cues, controls cell proliferation, differentiation and apoptosis. In metazoans, the protein Ras, Raf and MEK act sequentially to activate ERK [85]. Remarkably, we report that DVAP-P58S-mediated ALS phenotypes were sensitive to the dosage of a number of genes that function upstream of Raf and downstream of Ras including connector enhancer of KSR (CNK), Src42 and 14-3-3ζ. CNK functions as multivalent adaptor protein that cooperates with Ras and Src42 to induce activation of Raf [86,87].

Interestingly, we show that Hippo (Hpo) may be up-regulated in neurons expressing the *DVAP-P58S* transgene as reducing the genetic dosage of the tumor suppressor gene Hpo by using two independently generated alleles is sufficient to suppress the effect of DVAP-P58S expression on the eye neurodegenerative phenotype(S6 Table). Additionally, the same genetic manipulation fully rescued the locomotion defects associated with pan-neural expression of the pathogenic transgene. In agreement with these data, overexpression of Hpo represses cell proliferation and induces apoptosis [88] while the mammalian homologue MST1 is hyperactivated in ALS patients and its down-regulation delays disease onset and extends survival in SOD1 mice models for ALS [89].

It has been reported that Hpo promotes apoptosis by reducing the expression levels of a number of downstream targets including the *Drosophila* inhibitor of apoptosis 1 (DIAP1) [90]. We have previously shown that DIAP1 is down-regulated in DVAP-P58S mutant background and that up-regulation of DIAP1 mitigates DVAP-P58S neurodegeneration. These data provide further support to the hypothesis that in triggering neurodegeneration by apoptosis, DVAP-P58S acts via the Hpo tumor suppressor pathway [15]. Recently, a high throughput protein-protein interaction analysis looking for additional genetic members of the Hpo pathway identified DVAP as a high-confidence member of the Hpo interactome [91]. This study also implicated a fundamental role for the vesicular trafficking process in various aspects of the Hpo signaling and the importance of VAPB protein function in intracellular vesicular trafficking has been well documented [44,45]. These data reinforce our genetic evidence supporting a role for the tumour suppressor gene Hpo in ALS pathogenesis and provide a landscape of molecular interactions that can promote the formulation of additional mechanistic hypotheses to be experimentally validated. Intriguingly, hVAPB can act as an oncoprotein as overexpression of hVAPB in mammary epithelial cells induces an increase in cell proliferation and its expression levels are elevated in primary and metastatic tumor specimens [46].

Although further correspondence between the *Drosophila* model and the human condition remains to be determined, the relationship between DVAP-P58 phenotypes and the *Drosophila* modifiers, raises the possibility that pharmacological manipulation of these genes may assist in developing treatments to ameliorate or prevent ALS-related abnormalities.

Note added in proof. While our manuscript was under revision, Liu and co-workers [92] reported that reactive oxygen species due to mitochondrial dysfunction lead to LD accumulation, which, in turn, promotes neurodegeneration both in *Drosophila* and mice. These findings are in agreement with the data of our screen showing that one of the largest and most effective categories of DVAP-P58S modifiers is represented by genes involved in LD biogenesis and dynamics. Therefore these two works converge together in designating a role for LD dysfunction in the aetiology of ALS and, possibly, other neurodegenerative diseases.

## Materials and Methods

### Screening methods and *Drosophila* husbandry

A stock carrying *ey-GAL4* and *UAS-DVAP-P58S* on the second chromosome was established by conventional recombination methods and used as a tester line for the screen. Individual EP or EPgy2 lines were crossed to this line and the F1 progeny was tested for suppression or enhancement of the *DVAP-P58S*-derived small and rough eye phenotype. To prevent accidental and progressive accumulation of extragenic modifiers of the *DVAP-P58S* phenotype, the *ey-Gal4*,*DVAP-P58S* stock was maintained at 22°C or at 18°C degrees and in these conditions no *DVAP-P58S*-induced eye phenotype was visible in the stock. Putative candidates isolated from the screen were then maintained for the next round of analysis. To quantify the eye surface area images were analyzed with ImageJ software (ImageJ software, National Institute of Health, Bethesda, MD, USA). The phenotypic effect of doubling the dosage of the *DVAP-P58S* transgene was studied in *CyO+* progeny derived from the cross of *ey-Gal4*,*DVAP-P58S/CyO* flies with flies carrying the *DVAP-P58S* transgene in homozygosity.

To test the effect of overexpressing candidate genes in the motor system, EP or EPgy2 individual stocks were crossed to the tester line expressing the *elav-GAL4* driver and the *DVAP-P58S* transgene. For the genetic experiments in which the phenotypic analysis was performed in the larval imaginal discs, the tester line was balanced over a “green balancer”. GFP expression from the balancer chromosome was used to select larvae for dissection that do not contain the balancer and on which the phenotypic analysis was carried out. Individual strains from the EP and EPgy2 collections as well as from the P{Mae-UAS.6.11} collection were tested for their ability to genetically modify the *ey-Gal4*-*DVAP-P58S* eye phenotype by mating 8 to 10 males of their strain to 10–15 females of the *ey-GAL4*,*DVAP-P58S/CyO* screening stock. After 2 days, adults were transferred to a fresh vial to create a duplicate cross and to maintain optimal cultural density. For the same reason, adults were discarded from the duplicate vial after an additional 2 days had passed. Embryos from both vials were raised at a temperature of 30°C in a water bath to maximize the expression of the *Gal4*. For the analysis of the enhancing effect, modifiers were first identified because, when compared to the tester line, they exhibit a reduction in the eye size and organism viability at 30°C. The potential enhancing effect of these lines was subsequently confirmed by quantifying the decrease in eye size and viability at 28°C. After crossing females of the *ey*,*DVAP-P58S/CyO-GFP* line with wild-type Canton S males, the viability of the tester line (V_tes_) was calculated as a ratio between the number of *CyO* ^*+*^ *-GFP* flies (*ey*,*DVAP-P58S/+*) over the number of expected flies based on the Mendelian ratio. The viability of the *ey*,*DVAP-P58S* in the presence of the enhancer line (V_enh_) was calculated in the same way. The normalized lethality for every enhancer was expressed as (1-V_enh_/V_tes_) x 100.

RNAi lines were acquired from the Vienna *Drosophila* RNAi Center while the entire overexpressor collection and a few TRIP RNAi lines were from the Bloomington *Drosophila* stock Center.

### Functional annotation and association network analyses

We performed gene set enrichment analysis (GSEA) on the modifier list by first de-correlating annotated terms in the GO-graph using the weighted elimination method of [43] resulting in conditionally independent GO-terms. We tested for term enrichment by hypergeometric test and corrected for multiple testing by controlling the false discovery rate through application of the Benjamini Yekutieli correction (α = 0.05). The *Drosophila* association network was obtained by querying GeneMania with the modifier gene using all, but gene co-expression derived interactions. In order to determine whether the network produced contained summary features associated with real biological networks we compared the mean node-degree and mean node-betweenness of the modifier network with those of GeneMania networks constructed from 1000 randomly selected gene lists of the same size. We calculated Z-scores comparing these metrics between the modifier network and metric distributions from the random gene list derived networks. Human orthologues were mapped using predictions made by the DIOPT system. The modifier and the expanded hVAPB networks of the human orthologues were generated through the use of IPA (Ingenuity Systems at www.ingenuity.com). All networks were visualised using Cytoscape [93].

### Climbing assay

Females of genotype *elav-Gal4;DVAP-P58S* were crossed to males of the mutant strains. Climbing assays were performed on 10 age-matched adult female flies raised at 28°C as described in [94]. The flies placed in a plastic vial were tapped to the bottom of the vial and the number of flies above 8 cm line was counted after 15 seconds. A total of 10 trials were performed every 48 hours.

### Immunohistochemistry on *Drosophila* larval tissues

Third instar larvae were dissected in cold 1x PBS and fixed at room temperature (RT) for 10 min in Bouin’s fixative (15 Picric Acid:5 Formaldehyde:1 Acetic Acid). Samples were washed in 0.1% Triton-X100 in PBS (PBT) and blocked in PBT containing 10% NGS for 2 hours at RT before being incubated overnight at 4°C with primary antibodies. A rabbit Rab5 primary antibody (Abcam, ab18211) was used at a concentration of 1: 1000 and the same concentration was used for the DVAP primary antibody made in guinea pig. The anti-syntaxin 7 antibody was used at a concentration of 1:200. Primary antibodies were washed off with PBT at RT for two hours by changing PBT every 15 minutes. Samples were incubated with secondary antibodies at RT for 2h and washed for 2 hours in PBT with changes of PBT every 15 minutes. Secondary antibodies were purchased from Jackson ImmunoResearch and used at a concentration of 1:500. Stainings on NMJs were performed as previously described [15]. Tissues were mounted in Vectashield Mounting Media (Vector Laboratories). Preparations were imaged on a Zeiss Axiovert LSM510 confocal microscope.

### Immunohistochemistry on *post-mortem* human spinal cord tissues

Human tissue was obtained from the MRC Edinburgh Brain Bank with full ethical approval for research studies by EoSRES (East of Scotland Research Ethics Service), Ref. No. 11/ES/0022. For our analysis, we used 3 ALS cases and the same number of age-matched controls. The age range of ALS cases was 55–63, with 2 females and 1 male case. Disease duration from diagnosis to death ranged from 7 months to 22 months with an average of 13 months. All cases were sporadic and none of them showed the C9orf72 expansion. The age-matched controls died from cardiac disease and after full neuro-pathological *post-mortem* examination, were considered to have no neurological disease during life. Spinal cords from the cervical region were examined in all cases.

Human spinal cord tissue fixed in 10% neutral-buffered formalin was processed into paraffin. 7 μm sections were cut and de-paraffinized with xylene before being rehydrated through graded ethanol solutions. Sections were pre-treated using heat-induced epitope retrieval with Novocastra pH6 retrieval buffer in a decloaking chamber by heating to 125°C for 10sec, cooling to 90°C before washing in running tap water. For immuno-enzymatic stainings, slides were stained on a Leica Vision Biosystems Bond robot using the refine polymer detection kit (Leica) as follows. Endogenous peroxidase was blocked with 3% hydrogen peroxide in TBST for 10 min. Sections were incubated with rabbit anti-RAB5 (1:250, Bethyl Laboratories) primary antibody in 0.1% TBST for 2 hours at 25°C and then incubated with anti-rabbit HRP polymer for 15 minutes at 25°C. Staining was visualized using 3,3’-diaminobenzidine as chromogen. Tissue was finally subjected to haematoxylin staining, dehydrated through graded ethanol, cleared in xylene and mounted in Pertex (Cellpath).

For immuno-fluorescence stainings, following dewaxing in xylene and rehydration in graded ethanols, sections were rinsed in water and then subjected to heat-induced epitope retrieval by heating to 125°C in a decloaking chamber (Biomed) before passively cooling to 90°C in Novocastra pH 6 retrieval buffer. Sections were washed in running tap water then placed on a Leica Biosystems Bond X robot for dual tyramide staining as follows. Following blocking in 3% H_2_O_2_ for 30 mins in Bond wash buffer and 30 mins in 20% normal goat serum, sections were incubated with the RAB5 antibody at a 1:500 dilution for 60 mins. After two washes in Bond buffer for 5 mins, sections were incubated with goat anti-rabbit peroxidase Fab fragments (Abcam) for 30 mins. Sections were again washed twice for 5 mins in Bond buffer and incubated with Tyramide-Cy3 for 10 mins (PerkinElmer). After two additional washes in Bond buffer for 5 mins, sections were subjected to heat-induced antigen retrieval for a further 10 mins using Leica Bond ER1 solution on the robot. Following a wash in bond buffer and blocking in 3% H_2_O_2_ for 30 mins in Bond wash buffer and 30 mins in 20% normal goat serum, sections were incubated with an anti-hVAPB antibody at 1:1000 dilution for 60 mins. Sections were then incubated with goat anti-mouse peroxidase fab fragments (Abcam) for 30 mins and subsequently with Tyramide FITC for 10 mins (Perkin Elmer). Sections were mounted on slides using permafluor (Thermo Scientific) and examined on a Zeiss Axiovert LSM510 confocal microscope.

### Statistical analysis

Statistical analysis was performed and graphs were generated using GraphPad 5.0. For experiments with more than two samples, a one-way ANOVA test was applied. Tukey’s multiple comparison test was then used as a post-hoc test when a significant difference was found in the ANOVA test. For experiments with only two samples, a two-tailed unpaired Student’s t-test was applied. For the climbing assay data, two-way ANOVA and Bonferroni as a post-hoc test were used to compare differences in motor performance between genotypes at different time points.

## Supporting Information

S1 Fig DVAP-P58S induces eye degeneration in a dosage-dependent manner. (A-G) Stereomicroscope images of external eyes from control flies (*ey-Gal4/+*) and flies expressing one (*ey*,*DVAP-P58S*) or two (*ey*,*DVAP-P58S/DVAP-P58S*) copies of the *DVAP-P58S* transgene in the eye at 25°C, 28°C and 30°C. (H) Estimated eye surface areas of the indicated genotypes presented as scatter plots. Red lines represent the average surface area of flies of the specified genotypes raised at 25°C, blue lines for flies raised at 28°C and green lines for flies raised at 30°C. At 25°C a single copy of *DVAP-P58S* transgene induces a small but significant change in eye size, while a severe reduction is observed with two copies of the same allele. At higher temperatures, one copy of the transgene is sufficient to induce severe neurodegeneration in the eye, while expression of a double copy induces organism lethality. (I-M) Eye imaginal discs of control larvae (*ey-Gal4/+* in I), and larvae of the indicated genotypes incubated at the specified temperatures were immuno-stained with anti-DVAP antibodies. In controls, DVAP immuno-reactivity has a granular pattern distributed throughout the cytoplasm at any of the tested temperatures. Only control eye imaginal discs from larvae incubated at 30°C are shown. A double dose of *DVAP-P58S* at 25°C induces accumulation of aggregates similar to that caused by the expression of a single copy of the same transgene at higher temperatures. At 28°C, the accumulation of aggregates is less drastic than at 30°C, while a few sporadic inclusions are present in eye imaginal discs of larvae expressing only one copy of *DVAP-P58S* at 25°C. ***P<0.001. Scale bars: 50μm for eye images and 10μm for eye imaginal disc stainings.
(TIF)

S2 Fig Flow chart of the screen for genetic modifiers of DVAP-P58S-induced neurotoxicity and motor impairment. The screen process is depicted including the results of each screening step.
(TIF)

S3 Fig Abnormal accumulation of Avalanche (Syntaxin7) in *DVAP-P58S* eye imaginal discs. (A) Control (*ey-Gal4/+*) and (B) *DVAP-P58S* expressing eye imaginal discs (*ey*,*DVAP-P58S*) immuno-stained with anti-DVAP and anti-Avalanche (Avl) antibodies. Both proteins are homogenously distributed throughout the cytoplasm but Avl accumulates into aggregates partially overlapping with DVAP-positive inclusions in *DVAP-P58S* expressing cells. Scale bar: 10μm.
(TIF)

S4 Fig Strong suppressors of the motor disability associated with *DVAP-P58S* expression in neurons. Suppressors are classified as strong when exhibit a highly significant suppression over at least three tested time points. Blue lines represent the control line (*elav/+*), red lines indicate the *elav;DVAP-P58S* tester line and the black lines the *elav;DVAP-P58S* flies with the modifying gene in trans-heterozygosity. ***P<0.001, **P<0.01, *P<0.05.
(TIF)

S5 Fig Modifiers with an intermediate suppression effect on the motor disability associated with *DVAP-P58S* expression in neurons. Suppressors are classified as intermediate when they exhibit a significant effect over two time points. Blue lines represent the control line (*elav/+*), red lines indicate the *elav;DVAP-P58S* tester line and the black lines the *elav;DVAP-P58S* flies with the modifying gene in trans-heterozygosity. ***P<0.001, **P<0.01, *P<0.05.
(TIF)

S6 Fig Weak suppressors of the motor disability associated with *DVAP-P58S* expression in neurons. Suppressors are classified as weak when they exhibit a significant suppression effect only at one time point or a mild suppression effect over two time points. Blue lines represent the control line (*elav/+*), red lines indicate the *elav;DVAP-P58S* tester line and the black lines the *elav;DVAP-P58S* flies with the modifying gene in trans-heterozygosity. ***P<0.001, **P<0.01, *P<0.05.
(TIF)

S7 Fig Modifiers with an enhancing effect on the motor disability associated with *DVAP-P58S* expression in neurons. Blue lines represent the control line (*elav/+*), red lines indicate the *elav;DVAP-P58S* tester line and the black lines the *elav;DVAP-P58S* flies with the modifying gene in trans-heterozygosity. ***P<0.001, **P<0.01, *P<0.05.
(TIF)

S8 Fig Effect of neuronal expression of a subset of *DVAP-P58S* genetic modifiers on synaptic structure. (A-D) Representative confocal images of NMJs stained for HRP in *elav-Gal4/+* control (A), in *elav;Diap2* ^*G2326*^ (B), *elav;Ric* ^*G2693*^ (C) and in *elav;Sm* ^*EY07191*^ (D) larvae. (E) Quantification of bouton size at muscle 12 (type I and type III boutons) of abdominal segment 3 in *elav-Gal4/+* control, *elav;Diap2* ^*G2326*^, *elav;Ric* ^*G2693*^ and *elav;Sm* ^*EY07191*^ NMJs. (F) Quantification of total number of boutons on muscle 12 and 13 of abdominal segment 3 in *elav-Gal4/+* controls (275.3 ± 6.0, n = 8), *elav;Diap2* ^*G2326*^ (259.3 ± 4.8, n = 8), *elav;Ric* ^*G2693*^ (254.1 ± 4.8, n = 8) and *elav;Sm* ^*EY07191*^ (235.3 ± 3.7, n = 8) NMJs. Expression of *Ric* and *Sm* induces a small but statistically significant decrease in bouton number compared to controls. However, co-expression of *Ric* and *DVAP-P58S* induces a significant amelioration of *DVAP-P58S* synaptic phenotype while expression of *Sm* exacerbates the *DVAP-P58S* phenotype by leading to a severe disruption of the synaptic structural integrity. Scale bar: 10μm. Error bars denote SEM. *** P < 0.001, * P < 0.05, n.s. P > 0.05.
(TIF)

S9 Fig Overexpression of Rab11 suppresses the neurodegenerative phenotype associated with DVAP-P58S expression. Stereomicroscope images (A) and quantification of surface areas (B) of flies of the indicated genotypes. ***P<0.001. Scale bar: 50μm.
(TIF)

S10 Fig Expression of Rab5 in DVAP-P58S eye imaginal discs induces, at least in part, a normal Rab5 distribution. (A) Control *ey-Gal4/+* eye imaginal discs and (B) imaginal discs expressing either *DVAP-P58S* alone (*ey*,*DVAP-P58S*) or (C) *DVAP-P58S* together with *Rab5* (*ey*,*DVAP-P58S/Rab5* ^*EY10619*^) were stained with antibodies as indicated. While Rab5 is localized to small punctuate structures in controls, it accumulates and overlaps with DVAP-positive aggregates in *DVAP-P58S* eye imaginal discs. In discs in which *DVAP-P58S* and *Rab5* are simultaneously co-expressed, Rab5 localization appears to be, at least in part, normal. Scale bars: 10μm.
(TIF)

S1 Table List of suppressors recovered from the functional genetic screen. *Drosophila* full names and gene symbols are listed along with the allele used for each gene, its stock identification number and the symbol of the relative human orthologue. The degree of modifying activity is also reported for every suppressor. Known molecular activities associated with suppressors were identified using the PANTHER classification system (www.pantherdb.org).
(DOCX)

S2 Table List of genes with an enhancing activity. *Drosophila* full names and gene symbols together with the allele used for each specific gene, its stock identification number and the symbol of the corresponding human orthologue are reported. The scored phenotypes (eye, lethality) and the degree of modifying activity for every interacting gene is indicated. Known molecular activities for each enhancer were identified according to the PANTHER classification system (www.pantherdb.org).
(DOCX)

S3 Table Validation of the modifying activity of identified hits. *Drosophila* full names and gene symbols are reported together with the name of the new allele used, the validated phenotypes (eye, lethality or both) and the degree of suppression/enhancement effect. The effect of the identified modifiers was confirmed using an independent allele. The majority were confirmed by using an RNAi line from the VDRC stock Center and these alleles are indicated with a “v” preceding their line number. A few modifiers were confirmed by using a TRIP RNAi line and are indicated with a “t” preceding the ID stock number. Some genes were validated using a specific UAS line or an independent EP or EPgy2 overexpressor line. A few genes were confirmed using a loss-of-function allele while syntaxin 7 was confirmed using a specific antibody (S3 Fig). A list of unconfirmed modifiers is reported possibly due to an insufficient knockdown of the gene targeted by the selected RNAi line.
(DOCX)

S4 Table Seventy seven of the eight five modifiers have a human orthologue. *Drosophila* full names and gene symbols are reported for every genetic hit along with the gene symbols, full names and gene ID of the corresponding human orthologue. The DIOPT system used for homology searches relies on a combination of 10 different algorithms. Human orthologues identified by only one algorithm (DIOPT score < 2) are not reported.
(DOCX)

S5 Table Human orthologues of *DVAP-P58S* modifiers causing neurological disorders. Symbols of the *Drosophila* gene and its human orthologue are reported along with the name of the disease, the MIM number and the source of information (OMIM or GWAS).
(DOCX)

S6 Table Classification of DVAP-P58S modifiers according to validation tests and phenotypic readouts. (DOCX)
