# Supplementary figures and images for: Network Analyses Reveal Novel Aspects of ALS Pathogenesis
Source: PLoS Genet. 2015 Mar 31;11(3):e1005107. doi: 10.1371/journal.pgen.1005107 (PMC4380362; doi:10.1371/journal.pgen.1005107)

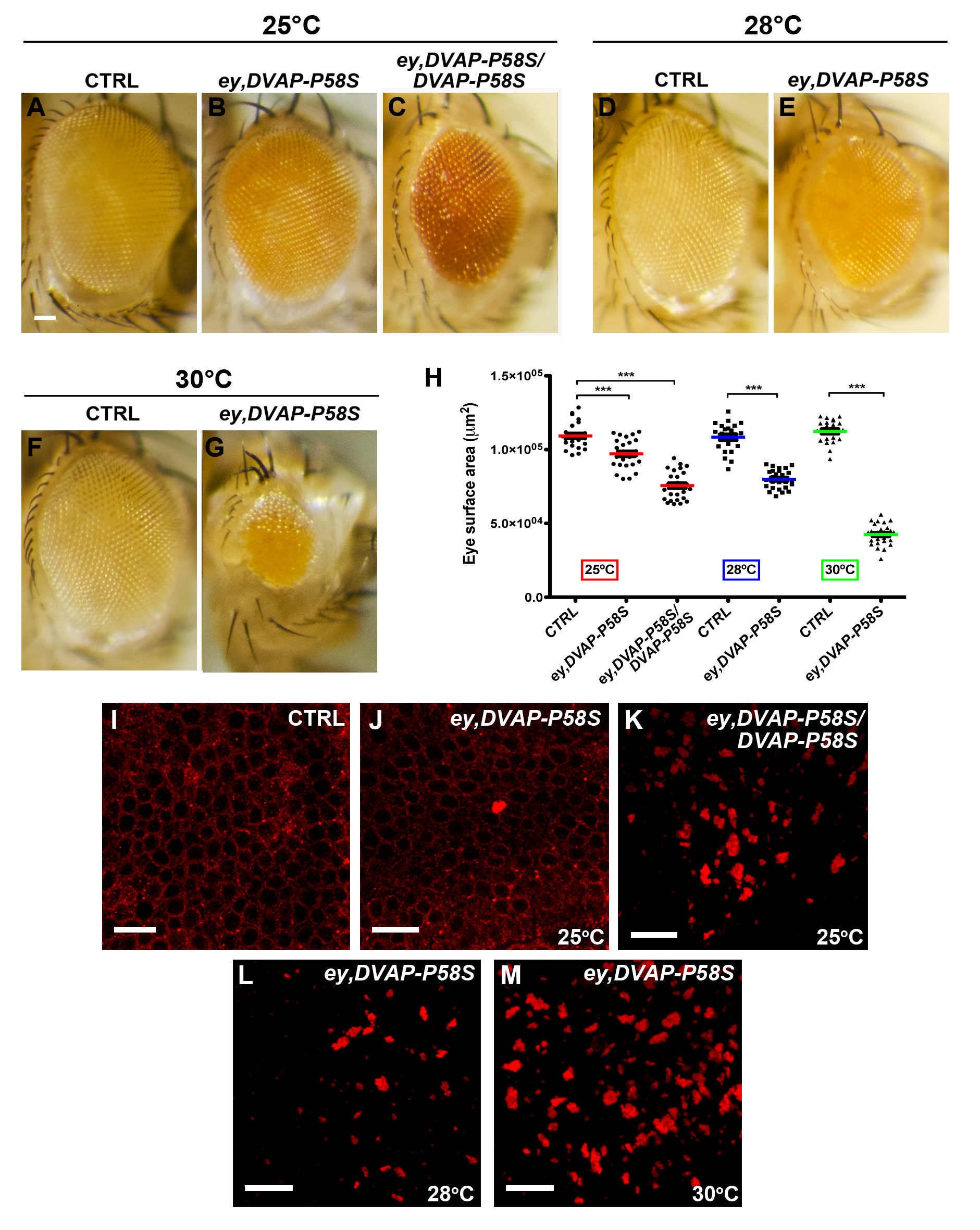

Supplement: S1 Fig — (A-G) Stereomicroscope images of external eyes from control flies (ey-Gal4/+) and flies expressing one (ey,DVAP-P58S) or two (ey,DVAP-P58S/DVAP-P58S) copies of the DVAP-P58S transgene in the eye at 25°C, 28°C and 30°C. (H) Estimated eye surface areas of the indicated genotypes presented as scatter plots. Red lines represent the average surface area of flies of the specified genotypes raised at 25°C, blue lines for flies raised at 28°C and green lines for flies raised at 30°C. At 25°C a single copy of DVAP-P58S transgene induces a small but significant change in eye size, while a severe reduction is observed with two copies of the same allele. At higher temperatures, one copy of the transgene is sufficient to induce severe neurodegeneration in the eye, while expression of a double copy induces organism lethality. (I-M) Eye imaginal discs of control larvae (ey-Gal4/+ in I), and larvae of the indicated genotypes incubated at the specified temperatures were immuno-stained with anti-DVAP antibodies. In controls, DVAP immuno-reactivity has a granular pattern distributed throughout the cytoplasm at any of the tested temperatures. Only control eye imaginal discs from larvae incubated at 30°C are shown. A double dose of DVAP-P58S at 25°C induces accumulation of aggregates similar to that caused by the expression of a single copy of the same transgene at higher temperatures. At 28°C, the accumulation of aggregates is less drastic than at 30°C, while a few sporadic inclusions are present in eye imaginal discs of larvae expressing only one copy of DVAP-P58S at 25°C. ***P<0.001. Scale bars: 50μm for eye images and 10μm for eye imaginal disc stainings. (TIF) [file pgen.1005107.s001.tif]

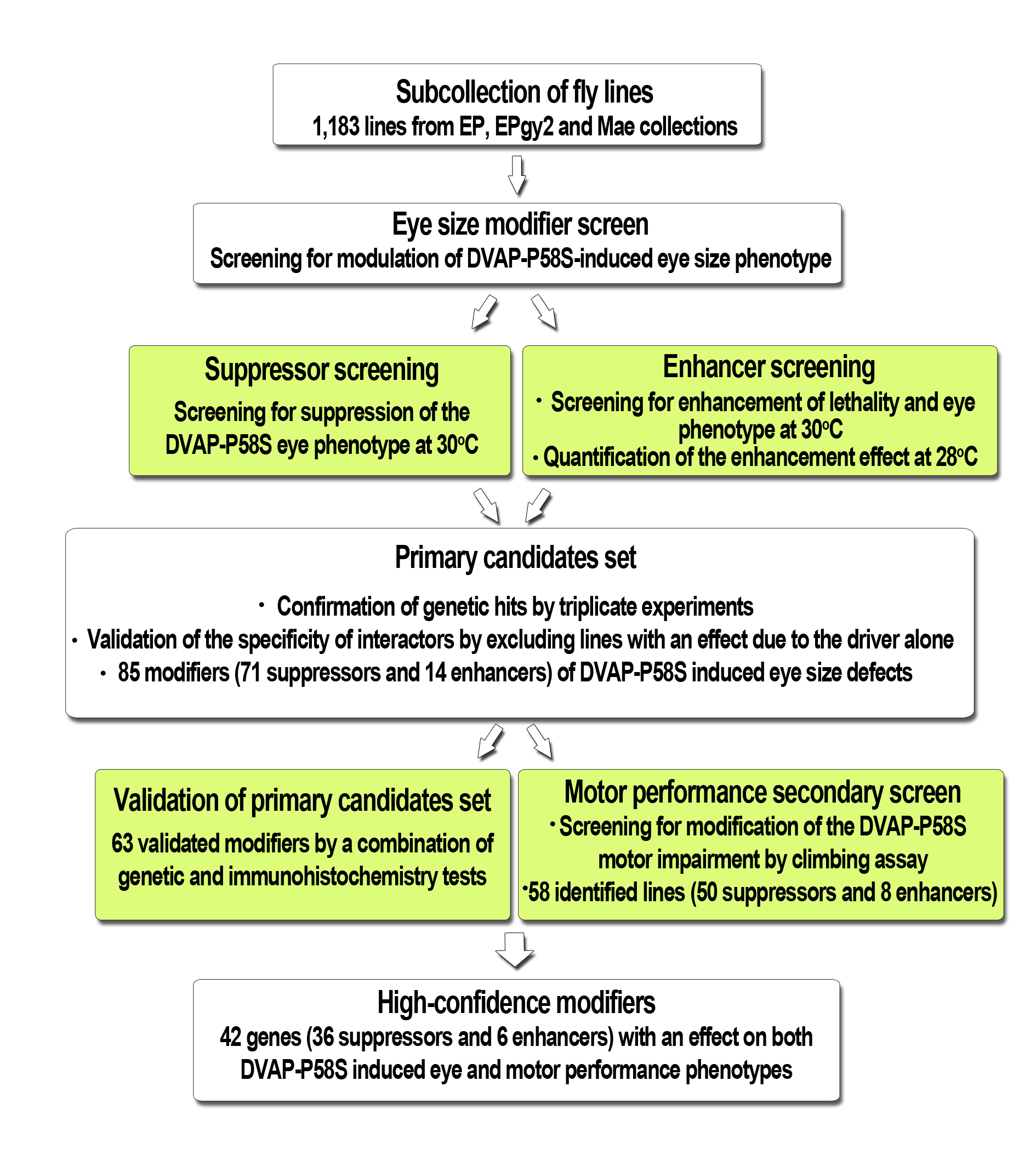

Supplement: S2 Fig — The screen process is depicted including the results of each screening step. (TIF) [file pgen.1005107.s002.tif]

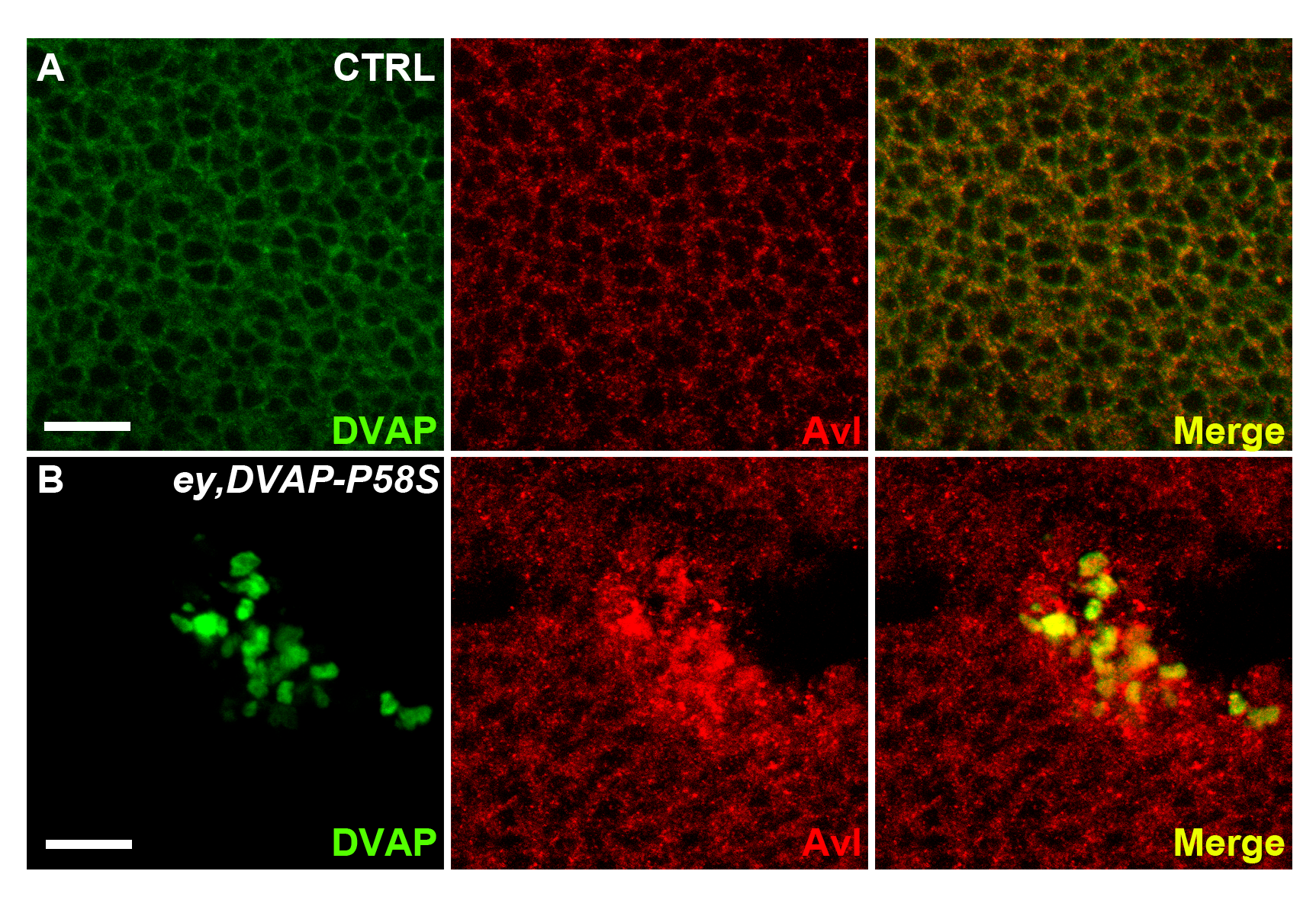

Supplement: S3 Fig — (A) Control (ey-Gal4/+) and (B) DVAP-P58S expressing eye imaginal discs (ey,DVAP-P58S) immuno-stained with anti-DVAP and anti-Avalanche (Avl) antibodies. Both proteins are homogenously distributed throughout the cytoplasm but Avl accumulates into aggregates partially overlapping with DVAP-positive inclusions in DVAP-P58S expressing cells. Scale bar: 10μm. (TIF) [file pgen.1005107.s003.tif]

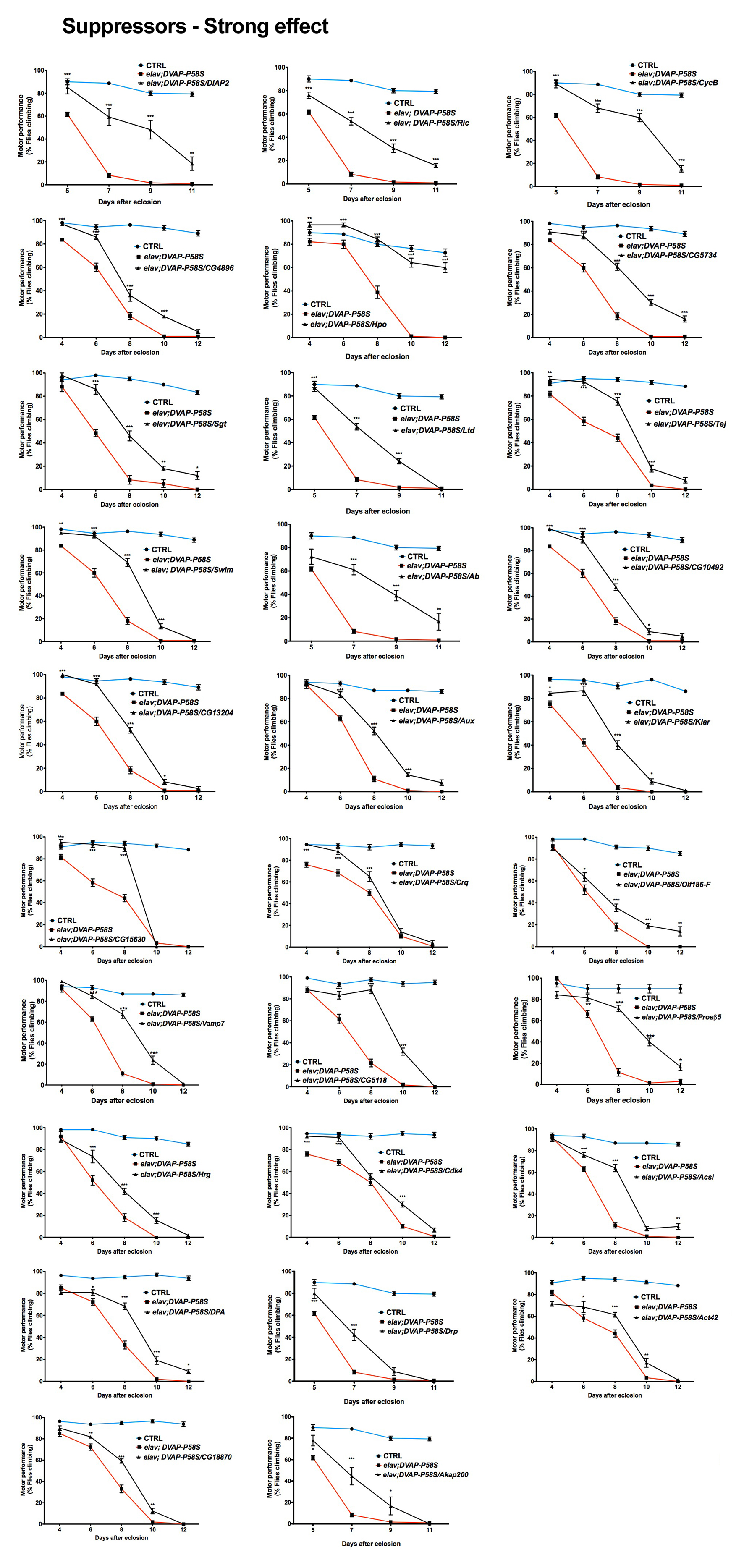

Supplement: S4 Fig — Suppressors are classified as strong when exhibit a highly significant suppression over at least three tested time points. Blue lines represent the control line (elav/+), red lines indicate the elav;DVAP-P58S tester line and the black lines the elav;DVAP-P58S flies with the modifying gene in trans-heterozygosity. ***P<0.001, **P<0.01, *P<0.05. (TIF) [file pgen.1005107.s004.tif]

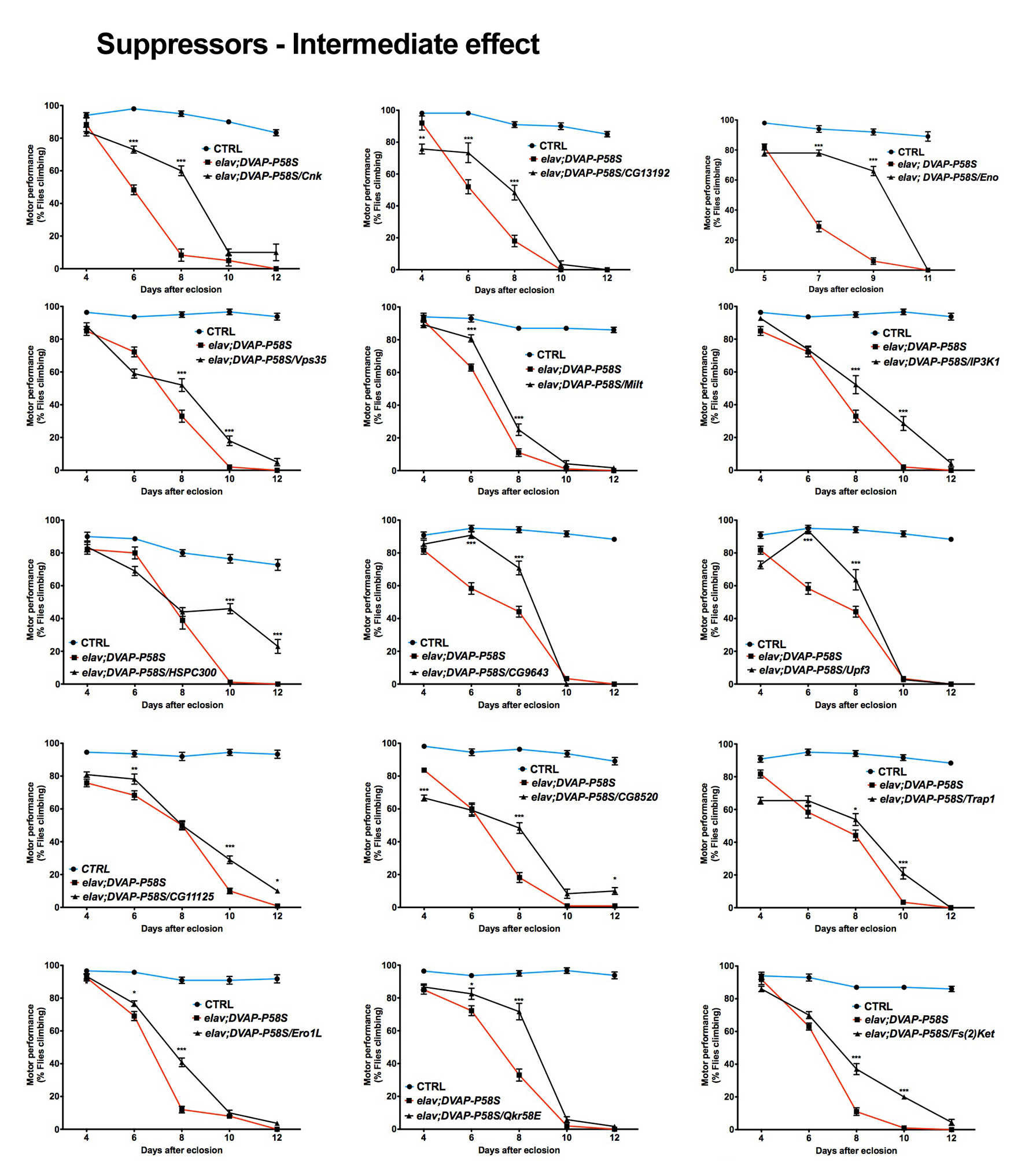

Supplement: S5 Fig — Suppressors are classified as intermediate when they exhibit a significant effect over two time points. Blue lines represent the control line (elav/+), red lines indicate the elav;DVAP-P58S tester line and the black lines the elav;DVAP-P58S flies with the modifying gene in trans-heterozygosity. ***P<0.001, **P<0.01, *P<0.05. (TIF) [file pgen.1005107.s005.tif]

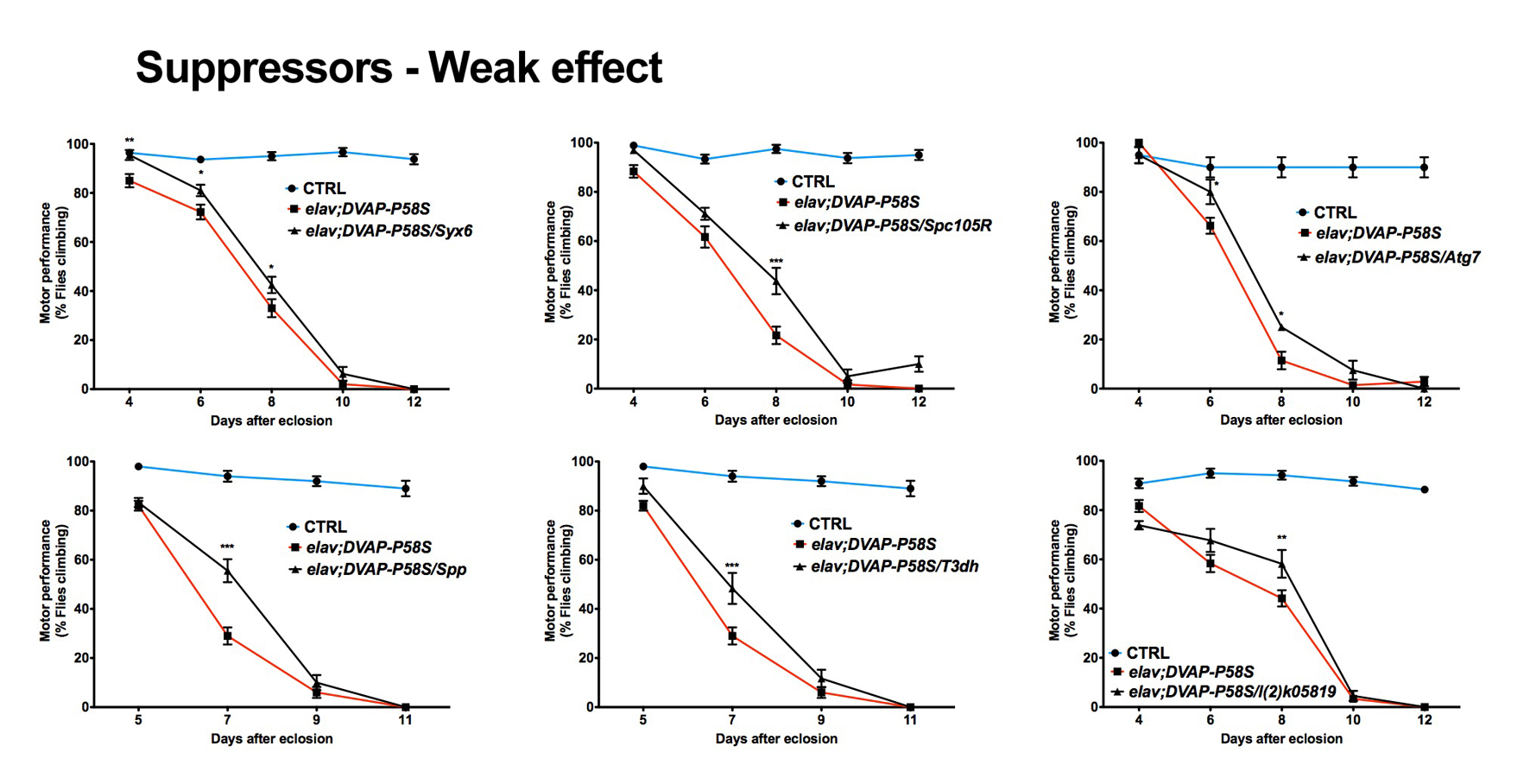

Supplement: S6 Fig — Suppressors are classified as weak when they exhibit a significant suppression effect only at one time point or a mild suppression effect over two time points. Blue lines represent the control line (elav/+), red lines indicate the elav;DVAP-P58S tester line and the black lines the elav;DVAP-P58S flies with the modifying gene in trans-heterozygosity. ***P<0.001, **P<0.01, *P<0.05. (TIF) [file pgen.1005107.s006.tif]

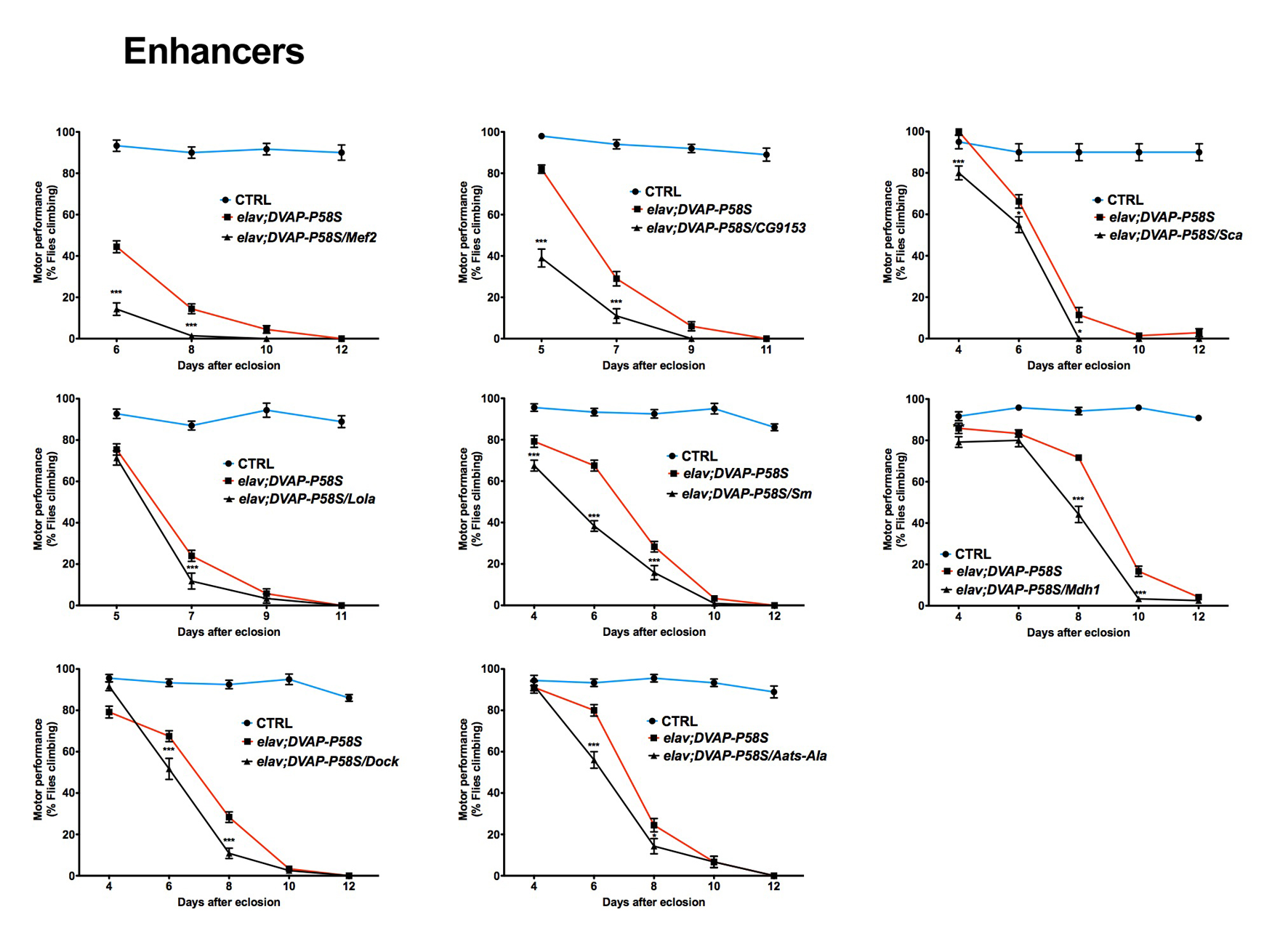

Supplement: S7 Fig — Blue lines represent the control line (elav/+), red lines indicate the elav;DVAP-P58S tester line and the black lines the elav;DVAP-P58S flies with the modifying gene in trans-heterozygosity. ***P<0.001, **P<0.01, *P<0.05. (TIF) [file pgen.1005107.s007.tif]

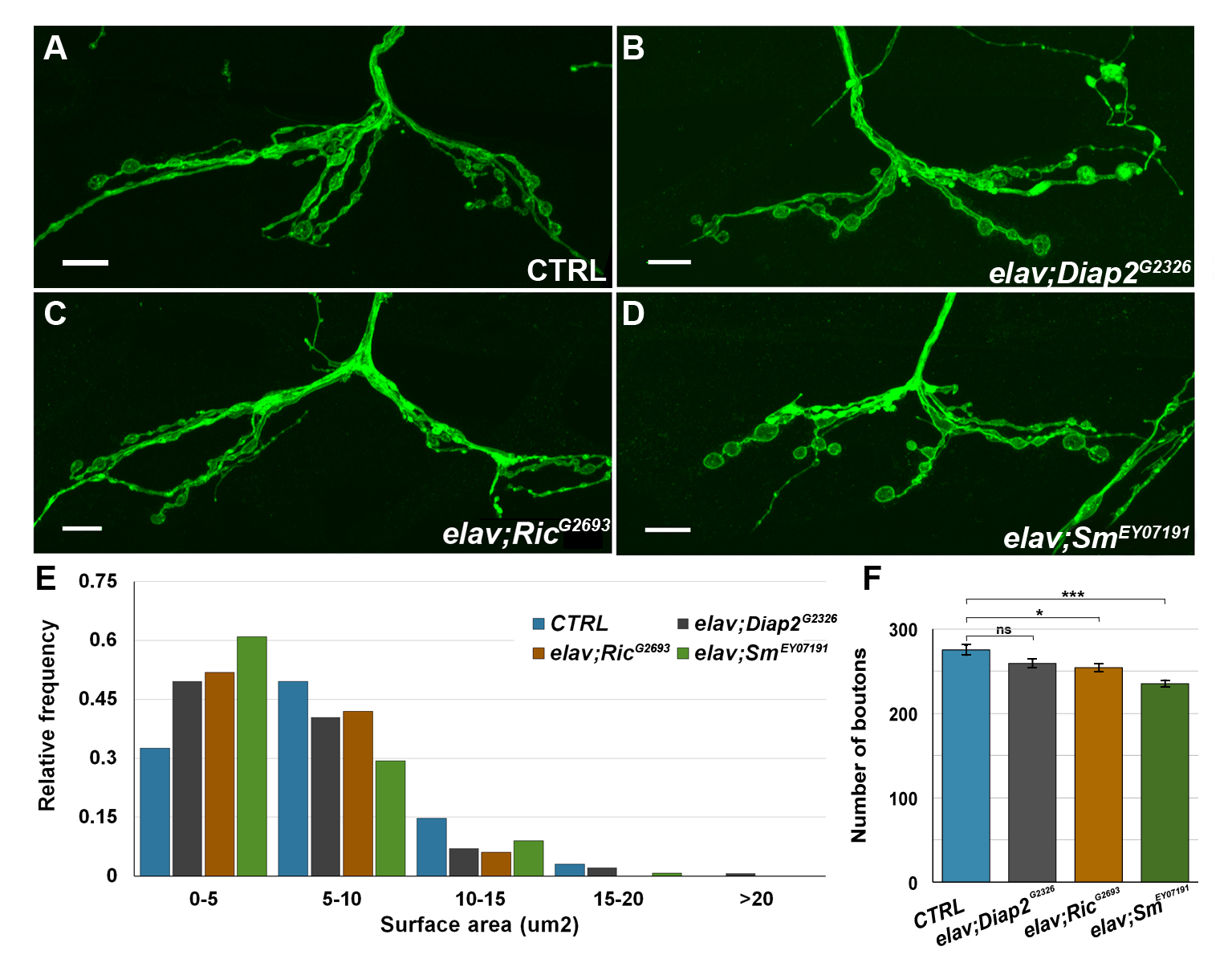

Supplement: S8 Fig — (A-D) Representative confocal images of NMJs stained for HRP in elav-Gal4/+ control (A), in elav;Diap2 G2326 (B), elav;Ric G2693 (C) and in elav;Sm EY07191 (D) larvae. (E) Quantification of bouton size at muscle 12 (type I and type III boutons) of abdominal segment 3 in elav-Gal4/+ control, elav;Diap2 G2326, elav;Ric G2693 and elav;Sm EY07191 NMJs. (F) Quantification of total number of boutons on muscle 12 and 13 of abdominal segment 3 in elav-Gal4/+ controls (275.3 ± 6.0, n = 8), elav;Diap2 G2326 (259.3 ± 4.8, n = 8), elav;Ric G2693 (254.1 ± 4.8, n = 8) and elav;Sm EY07191 (235.3 ± 3.7, n = 8) NMJs. Expression of Ric and Sm induces a small but statistically significant decrease in bouton number compared to controls. However, co-expression of Ric and DVAP-P58S induces a significant amelioration of DVAP-P58S synaptic phenotype while expression of Sm exacerbates the DVAP-P58S phenotype by leading to a severe disruption of the synaptic structural integrity. Scale bar: 10μm. Error bars denote SEM. *** P < 0.001, * P < 0.05, n.s. P > 0.05. (TIF) [file pgen.1005107.s008.tif]

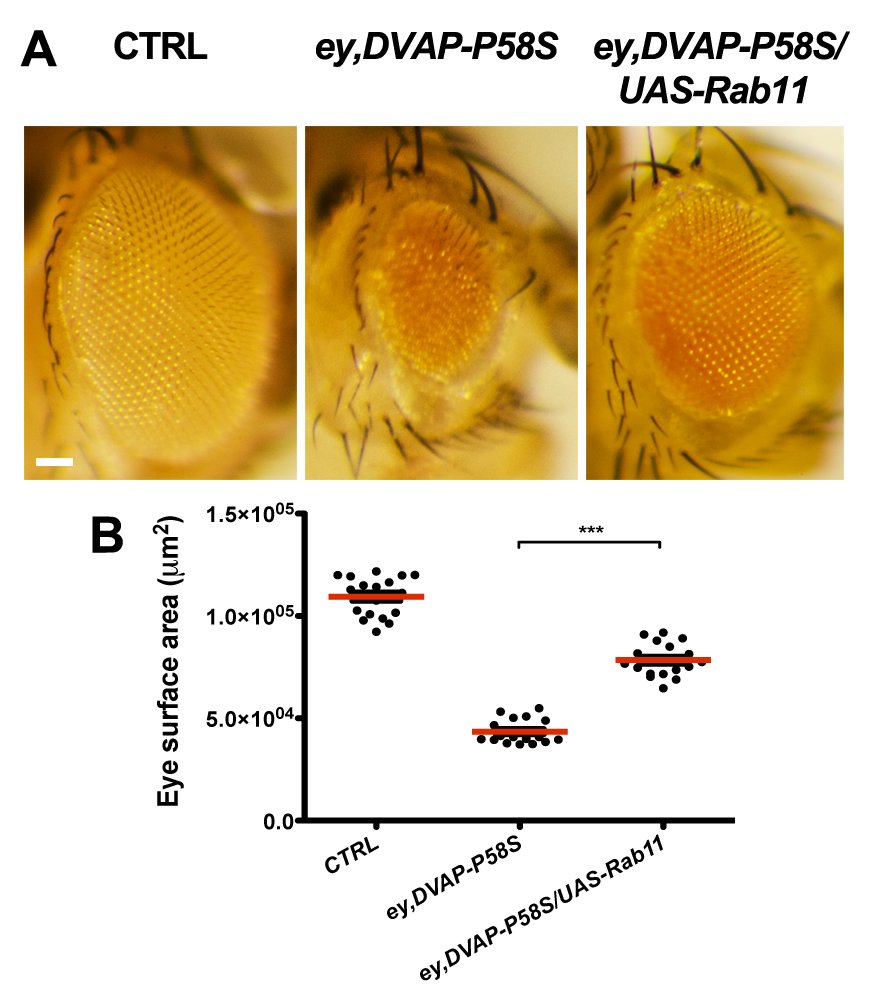

Supplement: S9 Fig — Stereomicroscope images (A) and quantification of surface areas (B) of flies of the indicated genotypes. ***P<0.001. Scale bar: 50μm. (TIF) [file pgen.1005107.s009.tif]

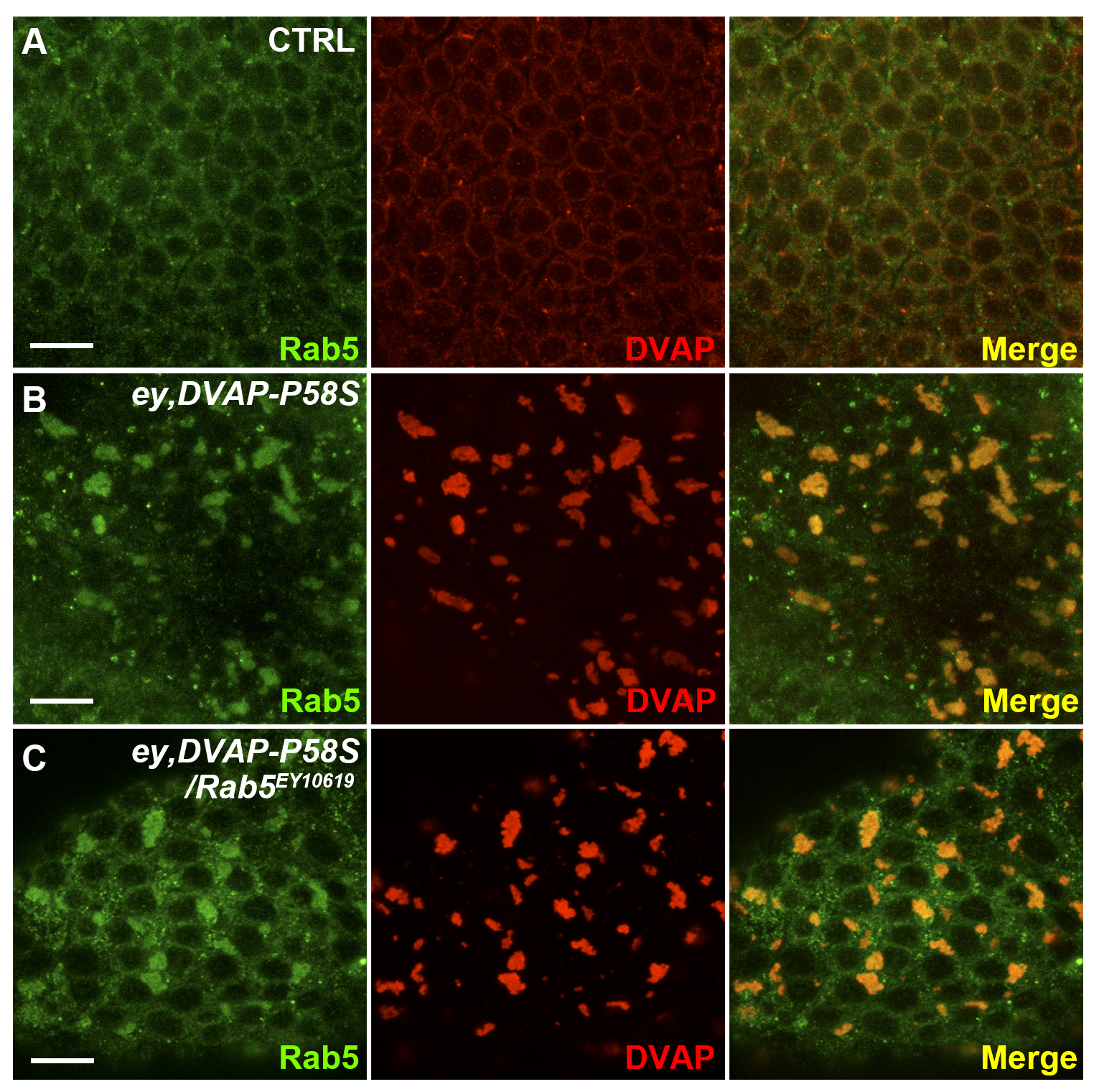

Supplement: S10 Fig — (A) Control ey-Gal4/+ eye imaginal discs and (B) imaginal discs expressing either DVAP-P58S alone (ey,DVAP-P58S) or (C) DVAP-P58S together with Rab5 (ey,DVAP-P58S/Rab5 EY10619) were stained with antibodies as indicated. While Rab5 is localized to small punctuate structures in controls, it accumulates and overlaps with DVAP-positive aggregates in DVAP-P58S eye imaginal discs. In discs in which DVAP-P58S and Rab5 are simultaneously co-expressed, Rab5 localization appears to be, at least in part, normal. Scale bars: 10μm. (TIF) [file pgen.1005107.s010.tif]
