# Supplementary material for: Network Analyses Reveal Novel Aspects of ALS Pathogenesis
Source: PLoS Genet. 2015 Mar 31;11(3):e1005107. doi: 10.1371/journal.pgen.1005107 (PMC4380362; doi:10.1371/journal.pgen.1005107)
Supplement: S1 Table — Drosophila full names and gene symbols are listed along with the allele used for each gene, its stock identification number and the symbol of the relative human orthologue. The degree of modifying activity is also reported for every suppressor. Known molecular activities associated with suppressors were identified using the PANTHER classification system (www.pantherdb.org). (DOCX) [file pgen.1005107.s011.docx]

| **Gene Name** | **Gene Symbol** | **Human ortholog** | **Modifying allele** | **Stock ID** | **% Sup** | **Protein class** |
| --- | --- | --- | --- | --- | --- | --- |
| Hormone receptor-like in 39 | Hr39 | NR6A1 | P{EPgy2}Hr39^EY04579^ | 20152 | 90.55 | Hormone receptor |
| Inhibitor of apoptosis 2 | Diap2 | BIRC2 | P{EP}Diap2^G2326^ | 26986 | 79.62 | Protease inhibitor |
| Small glutamine-rich  Tetratricopeptide containing protein | Sgt | SGTB | P{EPgy2}^EY02712^ | 15587 | 79.12 | Chaperone |
| CG5118 | CG5118 | - | P{EPgy2}CG5118^EY18569^ | 16541 | 76.58 | - |
| Spaghetti | Spag | RPAP3 | P{EPgy2}spag^EY11196^ | 20274 | 75.79 | Chaperone |
| Type III alcohol dehydrogenase | T3dh | ADHFE1 | P{EPgy2}T3dh^EY09338^ | 17559 | 74.65 | Dehydrogenase |
| Abrupt | ab | - | P{EPgy2}ab^EY09709^ | 16949 | 74.02 | Nucleic acid binding |
| Rap GTPase activating protein 1 | RapGAP1 | RAP1GAP2 | P{EPgy2}EY01137a | 15062 | 73.59 | G-protein modulator |
| Rhomboid | rho | RHBDL3 | P{EP}rho^EP3704^ | 17276 | 72.15 | Serine-protease |
| Upf3 | Upf3 | UPF3B | P{EPgy2}Upf3^EY03241^ | 16558 | 69.62 | Nucleic acid binding |
| Klarsicht | klar | - | P{EP}EP3104^EP3104^ | 6413 | 68.83 | Hydrolase |
| CG12299 | CG12299 | ZNF366 | P{EPgy2}CG12299^EY01579^ | 15520 | 68.45 | KRAB box transcription factor |
| Leak | lea | ROBO1 | P{EP}lea^EP2582^ | 17071 | 67.86 | Receptor |
| CG5734 | CG5734 | SNX17 | P{EPgy2}CG5734^EY05560^ | 15456 | 67.76 | Phosphatidyl inositol binding |
| Acyl-CoA synthetase long-chain | Acsl | ACSL3 | P{EPgy2}Acsl^EY07112^ | 19860 | 66.87 | Ligase |
| Actin 42A | Act42A | ACTB | P{EPgy2}Act42A^EY05608^ | 15460 | 66.67 | Motor protein |
| Tejas | tej | TDRD5 | P{EPgy2}tej^EY20088^ | 22361 | 66.19 | Signal transduction |
| CG15630 | CG15630 | NCAM1 | P{EPgy2}EY20668 | 22414 | 66.18 | Cell adhesion family |
| Inositol 1,4,5-triphosphate kinase 1 | IP3K1 | ITPKA | P{Mae-UAS.6.11}IP3K1^UY530^ | 6769 | 65.77 | Kinase |
| Costa | cos | KIF7 | P{EPgy2}cos^EY08735^ | 19759 | 65.64 | Motor protein |
| CG13204 | CG13204 | - | P{EPgy2}CG13204^EY11838^ | 21076 | 65.03 | Nucleic acid binding |
| Retinal degeneration B beta | rdgBβ | PITPNC1 | P{EP}rdgBβ^G8057^ | 27975 | 64.37 | Transporter |
| Suppressor of variegation 2-10 | Su(var)2-10 | PIAS1 | P{EPgy2}Su(var)2-10^EY01453^ | 19642 | 64.25 | Ligase |
| 14-3-3ζ | 14-3-3ζ | YWHAZ | P{EPgy2}14-3-3ζ^EY06147^ | 19919 | 63.42 | Chaperone |
| Vacuolar protein sorting 35 | Vps35 | VPS35 | P{EPgy2}Vps35^EY14200^ | 20913 | 63.20 | Membrane trafficking protein |
| Spc105-Related | Spc105-R | - | P{EP}G4635 | 28884 | 63.09 | - |
| CG3625 | CG3625 | AIG1 | P{EPgy2}CG3625^EY07089^ | 16406 | 62.88 | - |
| Milton | milt | TRAK1 | P{Mae-UAS.6.11}milt^LA00951^ | 22198 | 62.52 | Mitochondrial trafficking protein |
| Signal peptide peptidase | spp | HM13 | P{EP}G2086 | 27455 | 62.40 | Protease |
| Syntaxin 6 | Syx6 | STX6 | P{EPgy2}Syx6^EY14508^ | 20941 | 61.91 | SNARE protein |
| Silent information regulator 2 | Sir2 | SIRT1 | P{EP}Sir2^EP2300^ | 24859 | 61.43 | Deacetylase |
| Female sterile (2) Ketel | Fs(2)Ket | KPNB1 | P{EPgy2}Fs(2)Ket^EY06666^ | 15967 | 60.73 | Transporter |
| Lethal (2) k05819 | l(2)k05819 | KIAA0195 | P{EPgy2}EY03384 | 20150 | 60.55 | - |
| Enolase | Eno | ENO1 | P{EPgy2}Eno^EY23161^ | 22610 | 59.08 | Enolase |
| Hippo | hpo | STK3 | P{EP}hpo^G3315^ | 27105 | 58.58 | Kinase |
| CG10809 | CG10809 | ANKRD54 | P{EP}CG10809^G4564^ | 27161 | 58.17 | - |
| Ras which interacts with calmodulin | Ric | RIT2 | P{EP}Ric^G2693^ | 27003 | 58.10 | Small GTPase |
| Quaking related 58E-3 | qkr58E-3 | KHDRBS1 | P{EPgy2}qkr58E-3^EY02038^ | 15086 | 57.68 | Transcription factor |
| Vesicle-associated membrane protein 7 | Vamp7 | VAMP7 | P{EP}Vamp7^G7738^ | 28488 | 57.56 | SNARE protein |
| Cyclin-dependent kinase 4 | Cdk4 | CDK6 | P{EPgy2}Cdk4^EY09330^ | 19891 | 57.55 | Kinase |
| CG18870 | CG18870 | - | P{EPgy2}CG18870^EY06926^ | 15981 | 57.14 | - |
| Secreted Wg-interacting molecule | Swim | TINAGL1 | P{EPgy2}Swim^EY09931^ | 17622 | 56.49 | Protease |
| CG4896 | CG4896 | RBM5 | P{EPgy2}CG4896^EY01431^ | 19814 | 55.85 | - |
| CG9643 | CCG9643 | METTL10 | P{EPgy2}CG9643^EY07345^ | 16815 | 55.72 | Methyltransferase |
| Pepsinogen-like | Pcl | PGA3 | P{EPgy2}Pcl^EY08457^ | 19876 | 55.49 | Nucleic acid binding |
| CG8520 | CG8520 | LACE1 | P{EPgy2}CG8520^EY09481^ | 17573 | 55.43 | Hydrolase |
| Disc proliferation abnormal | dpa | MCM4 | P{EPgy2}dpa^EY04015^ | 15922 | 54.86 | DNA helicase |
| Ero1-like protein | Ero1L | ERO1LB | P{EP}Ero1L^G18511^ | 29300 | 54.83 | Oxidoreductase |
| Olf186-F | olf186-F | ORAI1 | P{EPgy2}olf186-F^EY01467^ | 20119 | 54.67 | Signaling molecule |
| Autophagy-specific gene 7 | Atg7 | ATG7 | P{EPgy2}Sec6^EY10058^ | 17635 | 54.22 | Transfer protein ligase |
| CG13192 | CG13192 | GNB1L | P{EPgy2}CG13192^EY07746^ | 17400 | 54.20 | G-protein coupled receptor |
| Ubiquitin-conjugating enzyme  E2Q-like | CG4502 | UBE2QL1 | P{EPgy2}CG4502^EY07938^ | 17415 | 53.95 | Ligase |
| Cullin-2 | Cul-2 | CUL2 | P{EPgy2}Cul-2^EY09124^ | 19883 | 52.79 | Ubiquitin-protein ligase |
| CG10492 | CG10492 | ZCCHC2 | P{EPgy2}CG10492^EY10125^ | 17640 | 52.75 | - |
| Croquemort | crq | SCARB1 | P{EPgy2}crq^EY14489^ | 20939 | 52.71 | Receptor |
| CG11125 | CG11125 | ENKD1 | P{EP}CG11125^G18969^ | 26951 | 52.37 | - |
| Auxillin | Aux | GAK | P{EP}aux^G6787^ | 30174 | 51.85 | Transcription factor |
| Rab5 | Rab5 | RAB5A | P{EPgy2}Rab5^EY10619^ | 20193 | 51.73 | Membrane trafficking  regulatory protein |
| Syntaxin 7 | Syx7 | STX7 | P{EP}Syx7^G6457^ | 28476 | 50.75 | SNARE protein |
| Syntaxin Interacting Protein 1 | HSPC300 | BRK1 | P{EP}HSPC300^G19021^ | 26953 | 50.54 | - |
| Trap1 | Trap1 | TRAP1 | P{EPgy2}Trap1^EY10238^ | 19974 | 50.43 | Chaperone |
| Draper | drpr | MEGF11 | P{EP}drpr^EP522^ | 17175 | 50.41 | Receptor/signaling molecule |
| Kismet | kis | CHD7 | P{EPgy2}kis^EY12846^ | 21391 | 49.16 | DNA helicase |
| Hiiragi | hrg | PAPOLG | P{EPgy2}hrg^EY10340^ | 17668 | 49.15 | Adenylyltransferase |
| A kinase anchor protein 200 | Akap200 | - | P{EP}Akap200^EP2254^ | 17037 | 48.14 | Kinase |
| Cyclin B | CycB | CCNB1 | P{EPgy2}CycB^EY08217^ | 19870 | 45.23 | Kinase activator |
| Src oncogene at 42A | Src42A | FRK | P{EPgy2}Src42A^EY08937^ | 19763 | 44.66 | Transmembrane receptor |
| Proteasome subunit beta 5 | Prosβ5 | PSMB5 | P{EPgy2}EY00934 | 14870 | 43.98 | Proteasome |
| Coronin | coro | CORO1C | P{EP}coro^GE15547^ | 26894 | 43.51 | Non-motor actin binding |
| Connector enhancer of ksr | cnk | CNKSR2 | P{EPgy2}cnk^EY06675^ | 20160 | 40.89 | Kinase modulator |
| Lightoid | ltd | RAB32 | P{EPgy2}ltd^EY07166^ | 20166 | 30.51 | GTPase activity |
