# Supplementary material for: Network Analyses Reveal Novel Aspects of ALS Pathogenesis
Source: PLoS Genet. 2015 Mar 31;11(3):e1005107. doi: 10.1371/journal.pgen.1005107 (PMC4380362; doi:10.1371/journal.pgen.1005107)
Supplement: S2 Table — Drosophila full names and gene symbols together with the allele used for each specific gene, its stock identification number and the symbol of the corresponding human orthologue are reported. The scored phenotypes (eye, lethality) and the degree of modifying activity for every interacting gene is indicated. Known molecular activities for each enhancer were identified according to the PANTHER classification system (www.pantherdb.org). (DOCX) [file pgen.1005107.s012.docx]

| **Gene Name** | **Gene Symbol** | **Human ortholog** | **Modifying allele** | **Stock ID** | **%Enhancement** | | **Protein class** |
| --- | --- | --- | --- | --- | --- | --- | --- |
|  |  |  |  |  | **Eye** | **Lethality** |  |
| Scabrous | sca | FGA | P{EPgy2}EY00639 | 20095 | 57.14 | 41.18 | Signaling molecule |
| Myocyte-specific enhancer factor 2 | Mef2 | MEF2A | P{EP}Mef2^EP2002a^ | 17230 | 40.80 | 58.77 | Transcription factor |
| Peroxisome biogenesis factor 10 | Pex10 | PEX10 | P{EP}Pex10^G5094^ | 27176 | 24.59 | 53.24 | Transporter |
| Smooth | sm | HNRNPL | P{EPgy2}sm^EY07191^ | 19727 | 23.01 | 20.30 | Splicing factor |
| CG9153 | CG9153 | HERC4 | P{EP}CG9153^G5486^ | 27181 | 22.60 | 47.63 | Ubiquitin-protein ligase |
| Mitochondrial carrier homolog 1 | Mtch | MTCH2 | P{EP}Mtch^G8642^ | 27981 | 20.49 | 46.35 | Mitochondrial carrier protein |
| CG7324 | CG7324 | TBC1D9 | P{EP}CG7324^G8800^ | 27480 | 20.07 | 60.05 | Hydrolase |
| Malate dehydrogenase | Mdh1 | MDH1 | P{EPgy2}Mdh1^EY08761^ | 16435 | 18.04 | 29.28 | Dehydrogenase |
| Calcium-binding protein 1 | CaBP1 | - | P{EPgy2}CaBP1^EY12345^ | 20346 | 17.74 | 34.99 | Isomerase |
| Alanyl-tRNA synthetase | Aats-ala | ITSN1 | P{EP}Aats-ala^G3500^ | 27111 | 17.66 | 41.18 | RNA binding protein |
| Longitudinals lacking | lola | AARS | P{EPgy2}lola^EY10040^ | 27480 | 17.37 | 40.28 | Nucleic acid binding |
| Dynamin associated protein 160 | Dap160 | NCK1 | P{EP}Dap160^EP2543^ | 19582 | 14.70 | 28.93 | Membrane trafficking protein |
| Dreadlocks | dock | PLEKHG4 | P{EPgy2}EY08327 | 19871 | 12.87 | 24.47 | Signaling molecule |
| CG30456 | CG30456 | PDIA6 | P{EP}EP2185 | 17237 | 12.25 | 52.06 | Signaling molecule |
