# Supplementary material for: Network Analyses Reveal Novel Aspects of ALS Pathogenesis
Source: PLoS Genet. 2015 Mar 31;11(3):e1005107. doi: 10.1371/journal.pgen.1005107 (PMC4380362; doi:10.1371/journal.pgen.1005107)
Supplement: S3 Table — Drosophila full names and gene symbols are reported together with the name of the new allele used, the validated phenotypes (eye, lethality or both) and the degree of suppression/enhancement effect. The effect of the identified modifiers was confirmed using an independent allele. The majority were confirmed by using an RNAi line from the VDRC stock Center and these alleles are indicated with a “v” preceding their line number. A few modifiers were confirmed by using a TRIP RNAi line and are indicated with a “t” preceding the ID stock number. Some genes were validated using a specific UAS line or an independent EP or EPgy2 overexpressor line. A few genes were confirmed using a loss-of-function allele while syntaxin 7 was confirmed using a specific antibody (S3 Fig). A list of unconfirmed modifiers is reported possibly due to an insufficient knockdown of the gene targeted by the selected RNAi line. (DOCX) [file pgen.1005107.s013.docx]

**Table S3. Validation of modifying activity of the identified hits.**

| Validation of modifiers with a suppressor effect by using RNAi alleles | | | | |
| --- | --- | --- | --- | --- |
| Gene name | Gene symbol | Stock ID | Validation | % Enhancement |
| Disc proliferation abnormal | dpa | v44484 | Eye + Lethality | 36.45 ; 44.56 |
| Rab5 | Rab5 | t34832 | Eye + Lethality | 27.53 ; 78.97 |
| Acyl-CoA synthetase long-chain | Acsl | v3222 | Eye + Lethality | 24.33 ; 37.55 |
| Actin 42A | Act42A | v12456 | Eye + Lethality | 38.63 ; 39.26 |
| Kismet | kis | v46685 | Eye + Lethality | 14.31 ; 62.46 |
| Milton | milt | v41508 | Lethality | 33.88 |
| Vesicle-associated membrane protein 7 | Vamp7 | v13316 | Lethality | 39.27 |
| Croquemort | crq | v45883 | Lethality | 31.79 |
| Small glu-rich tetratricopeptide protein | sgt | v22002 | Lethality | 39.26 |
| CG3625 | CG3625 | v40855 | Lethality | 48.16 |
| Hormone receptor-like in 39 | Hr39 | v37694 | Lethality | 19.18 |
| CG13192 | CG13192 | v32157 | Lethality | 38.41 |
| Lethal (2) k05819 | l(2)k05819 | v13555 | Lethality | 36.74 |
| CG10809 | CG10809 | v38407 | Lethality | 38.75 |
| CG5734 | CG5734 | v43798 | Lethality | 34.05 |
| CG10492 | CG10492 | v12356 | Lethality | 32.82 |
| CG12299 | CG12299 | v102146 | Lethality | 16.95 |
| Quaking related 58E-3 | qkr58E-3 | v26242 | Lethality | 27.17 |
| Lightoid | ltd | v104348 | Lethality | 25.54 |
| 14-3-3ζ | 14-3-3ζ | v48724 | Lethality | 36.33 |
| Ero1-like protein | Ero1L | v51169 | Lethality | 39.29 |
| CG11125 | CG11125 | v18413 | Lethality | 46.71 |
| Cyclin B | CycB | v43772 | Lethality | 11.77 |
| Signal peptide peptidase | spp | v7247 | Lethality | 39.27 |
| Leak | lea | v11823 | Lethality | 13.52 |
| Autophagy-specific gene 7 | Atg7 | v27432 | Lethality | 39.28 |
| Suppressor of variegation 2-10 | Su(var)2-10 | v30709 | Lethality | 18.59 |
| Hiiragi | hrg | v42283 | Lethality | 36.48 |
| Upf3 | Upf3 | v31444 | Lethality | 30.71 |
| Secreted Wg-interacting molecule | swim | v6617 | Lethality | 38.42 |
| CG18870 | CG18870 | v3471 | Lethality | 34.51 |
| Src oncogene at 42A | Src42A | v17643 | Lethality | 28.55 |
| CG13204 | CG13204 | t31913 | Lethality | 14.28 |
| CG8520 | CG8520 | v36546 | Lethality | 19.41 |
| Spaghetti | spag | v31253 | Lethality | 31.59 |
| Cyclin-dependent kinase 4 | Cdk4 | v40576 | Lethality | 50.23 |
| Proteasome subunit beta 5 | Prosβ5 | v38659 | Lethality | 100.00 |
| Spc105-Related | Spc105-R | t35466 | Lethality | 100.00 |
| Vacuolar protein sorting 35 | Vps35 | v22180 | Eye | 17.53 |

| Validation of modifiers with a suppressor effect by a loss-of-function allele | | | | |
| --- | --- | --- | --- | --- |
| Gene name | Gene symbol | Allele | Validation | %Enhancement |
| Costa | cos | P{lacW}cos^k16101^ | Eye + Lethality | 26.62 ; 48.82 |
| Rap GTPase activating protein 1 | RapGAP1 | P{Mae-UAS.6.11} RapGAP1^LA00889^ | Eye + Lethality | 24.12 ; 34.15 |
| Type III alcohol dehydrogenase | T3dh | Mi{ET1}T3dh^MB09825^ | Lethality | 40.86 |

| Validation of modifiers with a suppressor effect by an UAS allele | | | | |
| --- | --- | --- | --- | --- |
| Gene name | Gene symbol | Allele | Validation | %Suppression |
| Inhibitor of apoptosis 2 | Diap2 | UAS-DIAP2 | Eye | 71.08 |
| Ras which interacts with calmodulin | Ric | UAS-Ric | Eye | 57.44 |
| Silent information regulator 2 | Sir2 | UAS-Sir2 | Eye | 77.32 |
| Rhomboid | rho | UAS-Rho | Eye | 69.77 |
| Draper | drpr | UAS-Drp | Eye | 50.81 |

| Validation of modifiers with a suppressor effect by an EP or EPgy2 line | | | | |
| --- | --- | --- | --- | --- |
| Gene name | Gene symbol | Allele | Validation | %Suppression |
| Retinal degeneration B beta | rdgBβ | P{EP}rdgBβ^EP2360^ | Eye | 84.30 |
| A kinase anchor protein 200 | Akap200 | P{EPgy2}Akap200^EY01150^ | Eye | 61.81 |
| Polycomblike | Pcl | P{EP}Pcl^GE15295^ | Eye | 50.60 |

| Validation of modifiers with a suppressor effect by immuno-staining | | | |
| --- | --- | --- | --- |
| Gene name | Antibody | Tissue | Reference |
| Syntaxin 7 | rabbit anti-syx7 | Brains - eye discs | Lu and Bilder, 2005 |

| Validation of modifiers with a suppressor effect caused by a loss–of-function | | | | |
| --- | --- | --- | --- | --- |
| Gene name | Gene symbol | Stock ID | Validation | %Suppression |
| Syntaxin Interacting Protein 1 | HSPC300 | t35051 | Eye | 46.34 |
| Hippo | hpo | t27661 | Eye | 90.73 |
| Klarsicht | klar | Klar ^YG3^ | Eye | 62.89 |

| Validation of modifiers with an enhancer effect by RNAi lines | | | | |
| --- | --- | --- | --- | --- |
| Gene name | Gene symbol | Stock ID | Validation | %Suppression |
| CG30456 | CG30456 | v21186 | Eye | 41.38 |
| Scabrous | sca | v44527 | Eye | 65.51 |
| Smooth | sm | v28117 | Eye | 27.84 |
| Dreadlocks | dock | v37524 | Eye | 60.97 |
| Calcium-binding protein 1 | CaBP1 | v43148 | Eye | 55.11 |
| Myocyte-specific enhancer factor 2 | Mef2 | v15550 | Eye | 38.19 |
| CG9153 | CG9153 | t37220 | Eye | 43.57 |

| Validation of modifiers with a enhancer effect by a loss-of-function allele | | | | |
| --- | --- | --- | --- | --- |
| Gene name | Gene symbol | Allele | Validation | %Suppression |
| Alanyl-tRNA synthetase | Aats-ala | P{EPgy2}Aats-ala^EY01137b^ | Eye | 73.59 |
| Dynamin associated protein 160 | Dap160 | ∆Dap160 | Eye | 66.82 |

| Unconfirmed modifiers suppressors | | |
| --- | --- | --- |
| Gene Name | Gene symbol | Stock ID |
| Coronin | coro | v44671 |
| Inositol 1,4,5-triph kinase 1 | IP3K1 | t35296 |
| Enolase | eno | t26300 |
| Auxillin | aux | v16182 |
| Trap1 | Trap1 | v108300 |
| Connector enhancer of ksr | cnk | v107746 |
| Cullin-2 | Cul-2 | v19297 |
| Syntaxin 6 | Syx6 | v1501 |
| CG15630 | CG15630 | v37842 |
| Female sterile (2) Ketel | Fs(2)ket | v22348 |
| Ub-conjugating enzyme E2Q-like | CG4502 | v34855 |
| CG4896 | CG4896 | v26652 |
| Tejas | tej | v24181 |
| Olf186-F | Olf186-F | v12221 |
| Abrupt | ab | v41005 |
| CG9643 | CG9643 | v24081 |
| CG5118 | CG5118 | v34963 |

| Unconfirmed modifiers enhancers | | |
| --- | --- | --- |
| Gene Name | Gene symbol | Stock ID |
| Malate dehyrogenase | Mdh1 | v27398 |
| Longitudinals lacking | lola | v12573 |
| Mitochondrial carrier homolog 1 | Mtch | t16644 |
| Peroxisome biogenesis factor 10 | Pex10 | v46613 |
| CG7324 | CG7324 | v31063 |
