# Supplementary material for: Network Analyses Reveal Novel Aspects of ALS Pathogenesis
Source: PLoS Genet. 2015 Mar 31;11(3):e1005107. doi: 10.1371/journal.pgen.1005107 (PMC4380362; doi:10.1371/journal.pgen.1005107)
Supplement: S4 Table — Drosophila full names and gene symbols are reported for every genetic hit along with the gene symbols, full names and gene ID of the corresponding human orthologue. The DIOPT system used for homology searches relies on a combination of 10 different algorithms. Human orthologues identified by only one algorithm (DIOPT score < 2) are not reported. (DOCX) [file pgen.1005107.s014.docx]

**Table S4. Seventy seven of the eight five modifiers have a human orthologue.**

| ***Drosophila* Gene Name** | ***Drosophila* Symbol** | **Human Symbol** | **DIOPT score** | **Human Gene Name** | **Human Gene ID** |
| --- | --- | --- | --- | --- | --- |
| Hormone receptor-like in 39 | Hr39 | NR6A1 | 2 | nuclear receptor subfamily 6, group A  member 1 | 2649 |
| Inhibitor of apoptosis 2 | Diap2 | BIRC2 | 9 | baculoviral IAP repeat containing 2 | 329 |
| Small glutamine-rich  tetratricopeptide containing protein | Sgt | SGTB | 10 | small glutamine-rich tetratricopeptide  repeat (TPR)-containing, alpha | 54557 |
| CG5118 | CG5118 | - | - | - | - |
| spaghetti | Spag | RPAP3 | 10 | RNA polymerase II associated protein 3 | 79657 |
| Type III alcohol dehydrogenase | T3dh | ADHFE1 | 10 | alcohol dehydrogenase, iron containing, 1 | 137872 |
| abrupt | ab | - | - | - | 9278 |
| Rap GTPase activating protein 1 | RapGAP1 | RAP1GAP2 | 6 | RAP1 GTPase activating protein 2 | 23108 |
| Rhomboid | rho | RHBDL3 | 5 | rhomboid veinlet-like 3 | 162494 |
| Upf3 | Upf3 | UPF3B | 10 | UPF3 regulator of nonsense transcripts  homolog B | 65109 |
| Klarsicht | klar | - | - | - | - |
| CG12299 | CG12299 | ZNF366 | 2 | zinc finger protein 366 | 167465 |
| Leak | lea | ROBO1 | 5 | roundabout 1 | 6091 |
| CG5734 | CG5734 | SNX17 | 10 | sorting nexin 17 | 9784 |
| Acyl-CoA synthetase long-chain | Acsl | ACSL3 | 9 | acyl-CoA synthetase long-chain family  member 3 | 2181 |
| Actin 42A | Act42A | ACTB | 6 | actin, beta | 60 |
| Tejas | tej | TDRD5 | 3 | tudor domain containing 5 | 1635 |
| CG15630 | CG15630 | NCAM1 | 2 | neural cell adhesion 1 | 4684 |
| Inositol 1,4,5-triphosphate kinase 1 | IP3K1 | ITPKA | 2 | Inositol-trisphosphate 3-kinase A | 3706 |
| Costa | cos | KIF7 | 4 | Kinesin family member 7 | 374654 |
| CG13204 | CG13204 | - | - | - | - |
| Retinal degeneration B beta | rdgBβ | PITPNC1 | 9 | phosphatidylinositol transfer protein  cytoplasmic 1 | 26207 |
| Suppressor of variegation 2-10 | Su(var)2-10 | PIAS1 | 9 | protein inhibitor of activated STAT, 1 | 8554 |
| 14-3-3ζ | 14-3-3ζ | YWHAZ | 9 | tyrosine 3-monooxygenase/tryptophan  5-monooxygenase activation protein, zeta | 7534 |
| Vacuolar protein sorting 35 | Vps35 | VPS35 | 10 | vacuolar protein sorting 35 homolog | 55737 |
| Spc105-Related | Spc105R | - | - | - | - |
| CG3625 | CG3625 | AIG1 | 10 | androgen-induced 1 | 51390 |
| Milton | milt | TRAK1 | 10 | trafficking protein, kinesin binding 1 | 22906 |
| Signal peptide peptidase | spp | HM13 | 9 | histocompatibility (minor) 13 | 81502 |
| Syntaxin 6 | Syx6 | STX6 | 10 | Syntaxin 6 | 10228 |
| Silent information regulator 2 | Sir2 | SIRT1 | 8 | sirtuin 1 | 23411 |
| Female sterile (2) Ketel | Fs(2)Ket | KPNB1 | 10 | karyopherin (importin) beta 1 | 3837 |
| lethal (2) k05819 | l(2)k05819 | KIAA0195 | 9 | KIAAO195 | 9772 |
| Enolase | Eno | ENO1 | 9 | enolase 1, (alpha) | 2023 |
| Hippo | hpo | STK3 | 9 | serine/threonine kinase 3 | 6788 |
| CG10809 | CG10809 | ANKRD54 | 6 | ankyrin repeat domain 54 | 129138 |
| Ras which interacts with calmodulin | Ric | RIT2 | 9 | Ras-like without CAAX 2 | 6014 |
| quaking related 58E-3 | qkr58E-3 | KHDRBS1 | 4 | KH domain containing, RNA binding,  signal transduction associated 1 | 10657 |
| Vesicle-associated membrane protein 7 | Vamp7 | VAMP7 | 9 | vesicle-associated membrane protein 7 | 6845 |
| Cyclin-dependent kinase 4 | Cdk4 | CDK6 | 9 | cyclin-dependent kinase 6 | 1021 |
| CG18870 | CG18870 | - | - | - | - |
| Secreted Wg-interacting molecule | Swim | TINAGL1 | 9 | tubule-interstitial nephritis antigen-like 1 | 64129 |
| CG4896 | CG4896 | RBM5 | 8 | RNA binding motif protein 5 | 10181 |
| CG9643 | CG9643 | METTL10 | 6 | methyltransferase like 10 | 399818 |
| Polycomblike | Pcl | MTF2 | 8 | Metal response element binding transcription factor 2 | 22823 |
| CG8520 | CG8520 | LACE1 | 10 | lactation elevated 1 | 246269 |
| disc proliferation abnormal | dpa | MCM4 | 9 | minichromosome maintenance complex  component 4 | 4173 |
| Ero1-like protein | Ero1L | ERO1LB | 7 | ERO1-like beta | 56605 |
| olf186-F | olf186-F | ORAI1 | 10 | ORAI calcium release-activated calcium modulator 1 | 84876 |
| Autophagy-specific gene 7 | Atg7 | ATG7 | 10 | autophagy related 7 | 10533 |
| CG13192 | CG13192 | GNB1L | 9 | guanine nucleotide binding protein, beta  polypeptide 1-like | 54584 |
| Ubiquitin-conjugating enzyme E2Q-like | CG4502 | UBE2QL1 | 7 | ubiquitin-conjugating enzyme  E2Q family-like 1 | 134111 |
| Cullin-2 | Cul-2 | CUL2 | 10 | cullin 2 | 8453 |
| CG10492 | CG10492 | ZCCHC2 | 2 | zinc finger, CCHC domain containing 2 | 54877 |
| Croquemort | crq | SCARB1 | 3 | scavenger receptor class B, member 1 | 949 |
| CG11125 | CG11125 | ENKD1 | 7 | enkurin domain containing 1 | 84080 |
| Auxilin | Aux | GAK | 10 | cyclin G associated kinase | 2580 |
| Rab5 | Rab5 | RAB5A | 8 | RAB5A, member RAS oncogene family | 5868 |
| Syntaxin 7 | Syx7 | STX7 | 9 | syntaxin 7 | 8417 |
| Syntaxin Interacting Protein 1 | HSPC300 | BRK1 | 6 | BRICK1, SCAR/WAVE actin-nucleating  complex subunit | 55845 |
| Trap1 | Trap1 | TRAP1 | 10 | TNF receptor-associated protein 1 | 10131 |
| Draper | drpr | MEGF11 | 9 | multiple EGF-like-domains 11 | 84465 |
| kismet | kis | CHD7 | 9 | chromodomain helicase DNA binding protein 6 | 55636 |
| hiiragi | hrg | PAPOLG | 8 | poly(A) polymerase gamma | 64895 |
| A kinase anchor protein 200 | Akap200 | - | - | - | - |
| Cyclin B | CycB | CCNB1 | 6 | cyclin B1 | 891 |
| Src oncogene at 42A | Src42A | FRK | 9 | Fyn-related kinase | 2444 |
| Proteasome subunit beta 5 | Prosβ5 | PSMB5 | 10 | proteasome (prosome, macropain)  subunit beta, type, 5 | 5693 |
| Coronin | coro | CORO1C | 8 | coronin, actin binding protein, 1C | 23603 |
| connector enhancer of ksr | cnk | CNKSR2 | 7 | connector enhancer of kinase suppressor of Ras 2 | 22866 |
| Lightoid | ltd | RAB32 | 6 | member RAS oncogene family 32 | 10981 |
| scabrous | sca | FGA | 2 | fibrinogen alpha chain | 2243 |
| Myocyte-specific enhancer factor 2 | Mef2 | MEF2A | 7 | myocyte enhancer factor 2A | 4205 |
| Peroxisome biogenesis factor 10 | Pex10 | PEX10 | 10 | peroxisomal biogenesis factor 10 | 5192 |
| Smooth | sm | HNRNPL | 8 | heterogeneous nuclear ribonucleoprotein L | 3191 |
| CG9153 | CG9153 | HERC4 | 9 | HECT and RLD domain containing  E3 ubiquitin protein ligase 4 | 26091 |
| Mitochondrial carrier homolog 1 | Mtch | MTCH2 | 10 | mitochondrial carrier 2 | 23788 |
| CG7324 | CG7324 | TBC1D9 | 8 | TBC1 domain family, member 9 | 23158 |
| Malate dehyrogenase | Mdh1 | MDH1 | 10 | malate dehydrogenase 1, NAD (soluble) | 4190 |
| longitudinals lacking | lola | - | - | - | - |
| Dynamin associated protein 160 | Dap160 | ITSN1 | 9 | intersectin 1 (SH3 domain protein) | 6453 |
| Alanyl-tRNA synthetase | Aats-ala | AARS | 10 | alanyl-tRNA synthetase | 16 |
| Dreadlocks | dock | NCK1 | 10 | NCK adaptor protein 1 | 4690 |
| CG30456 | CG30456 | PLEKHG4 | 2 | pleckstrin homology domain containing,  family G, member 4 | 25894 |
| calcium-binding protein 1 | CaBP1 | PDIA6 | 10 | protein disulfide isomerase  family A, member 6 | 10130 |
