# Supplementary material for: Network Analyses Reveal Novel Aspects of ALS Pathogenesis
Source: PLoS Genet. 2015 Mar 31;11(3):e1005107. doi: 10.1371/journal.pgen.1005107 (PMC4380362; doi:10.1371/journal.pgen.1005107)
Supplement: S5 Table — Symbols of the Drosophila gene and its human orthologue are reported along with the name of the disease, the MIM number and the source of information (OMIM or GWAS). (DOCX) [file pgen.1005107.s015.docx]

**Table S5. Human orthologues of *Drosophila DVAP-P58S* modifiers causing neurological disorders.**

| **Human / *Drosophila***  Gene Symbol | Disease | **Phenotype**  MIM number | Source |
| --- | --- | --- | --- |
| UPF3B/Upf3 | Mental retardation, X-linked, syndromic 14 | 300676 | OMIM |
| ACTB/Act42A | Baraitser-Winter syndrome 1  Dystonia, juvenile-onset | 243310  607371 | OMIM  OMIM |
| KIF7/Cos | Acrocallosal syndrome  Joubert syndrome 12 Hydrolethalus syndrome 2 | 200990  200990  614120 | OMIM  OMIM  OMIM |
| PIAS1/Su(Var)2-10 | Major depressive disorder | - | GWAS |
| YWHAZ/14-3-3z | Attention deficits Hyperactivity disorder  Conduct disorder | -  -  - | GWAS GWAS GWAS |
| VPS35/Vps35 | Parkinson disease 17 | 614203 | OMIM |
| KPNB1/Fs(2)Ket | Multiple sclerosis | - | GWAS |
| STX6/Syx6 | Progressive supranuclear palsy | - | GWAS |
| RIT2/Ric | Cognitive performance  Parkinson’s disease | -  - | GWAS GWAS |
| MCM4/dpa | Natural killer cell and glucocorticoid deficiency with DNA repair defect | 609981 | OMIM |
| GAK/Aux | Parkinson’s disease | - | GWAS |
| CHD7/Kis | CHARGE syndrome | 214800 | OMIM |
| ENO1/eno | Autism spectrum disorder Attention deficit Schizophrenia | -  -  - | GWAS  GWAS  GWAS |
| FGA/sca | Amyloidosis, hereditary renal | 105200 | OMIM |
| PEX10/Pex10 | Peroxisome biogenesis disorder 1A Peroxisome biogenesis disorder 2B  Peroxisome biogenesis disorder 6A | 214100  202370  614870 | OMIM  OMIM  OMIM |
| AARS/Aats-ala | Charcot-Marie-Tooth disease, axonal, type 2N | 613287 | OMIM |
| PLEKHG4/CG9153 | Spinocerebellar ataxia 31 | 117210 | OMIM |
