# Supplementary material for: Network Analyses Reveal Novel Aspects of ALS Pathogenesis
Source: PLoS Genet. 2015 Mar 31;11(3):e1005107. doi: 10.1371/journal.pgen.1005107 (PMC4380362; doi:10.1371/journal.pgen.1005107)
Supplement: S6 Table — (DOCX) [file pgen.1005107.s016.docx]

**Table S6. Classification of DVAP-P58S modifiers according to genetic validation tests and to phenotypic readouts.**

| **Category** | **N** | **Modifiers** |
| --- | --- | --- |
| Validated modifiers with an effect on motor performance | 42 | Diap2, Sgt, T3dh, Upf3, klar, CG5734, Acsl, Act42A, Vps35, Spc105-R, spp, milt, Vamp7, l(2)k05819, hpo, Ric, qkr58E-3, Cdk4, CG18870, Swim, CG8520, hrg, dpa, Ero1L, Atg7, CG13192, CG10492, crq, CG11125, Akap200, HSPC300, drpr, CycB, Prosβ5, ltd, sca, sm, Mef2, Aats-ala, CG9153, dock, CG13204 |
| Unvalidated modifiers with an effect on motor performance | 16 | CG5118, ab, tej, IP3K1, CG15630, Syx6, Fs(2)Ket, CG9643, CG4896, olf186-F, Aux, Trap1, Eno, cnk, lola, Mdh1 |
| Validated modifiers with no effect on motor performance | 21 | Hr39, Spag, RapGAP1, rho, CG3625, CG12299, lea, cos, rdgBβ, Su(var)2-10,  14-3-3ζ, Sir2, CG10809, Pcl, Rab5, Syx7, kis, Src42A,CaBP1, Dap160, CG30456 |
| Modifiers of the eye neurodegenerative phenotype | 6 | CG4502, Cul-2, coro, CG7324, Mtch, Pex10 |
